# Overview of hepatocellular carcinoma: from molecular aspects to future therapeutic options

**DOI:** 10.1080/19336918.2023.2258539

**Published:** 2023-09-19

**Authors:** Sugan Panneerselvam, Cornelia Wilson, Prem Kumar, Dinu Abirami, Jayakrishna Pamarthi, Mettu Srinivas Reddy, Joy Varghese

**Affiliations:** a Department of Hepatology and Transplant Hepatology, Gleneagles Global Health City, Chennai, Tamil Nadu, India; b Natural and Applied Sciences, School of Psychology and Life Sciences, Canterbury Christ Church University, Discovery Park, Sandwich, UK; c Department of Gastroenterology, Gleneagles Global Health City, Chennai, Tamil Nadu, India; d Multi-Disciplinary Research Unit, Madras Medical College, Chennai, Tamil Nadu, India; e The Director and Head, Liver Transplant and HPB surgery, Gleneagles Global Health City, Chennai, Tamil Nadu, India

**Keywords:** Circulating tumour cell, HCC biomarkers, immunotherapy, cell signaling pathways, multi-omics technology

## Abstract

Hepatocellular carcinoma (HCC) is the seventh most highly prevalent malignant tumor globally and the second most common cause of mortality. HCC develops with complex pathways that occur through multistage biological processes. Non-alcoholic fatty liver disease, metabolic-associated fatty liver disease, alcoholic liver disease, autoimmune hepatitis, hepatitis B, and hepatitis C are the causative etiologies of HCC. HCC develops as a result of epigenetic changes, protein-coding gene mutations, and altered signaling pathways. Biomarkers and potential therapeutic targets for HCC open up new possibilities for treating the disease. Immune checkpoint inhibitors are included in the treatment options in combination with molecular targeted therapy.

## Introduction

**Figure 1.**
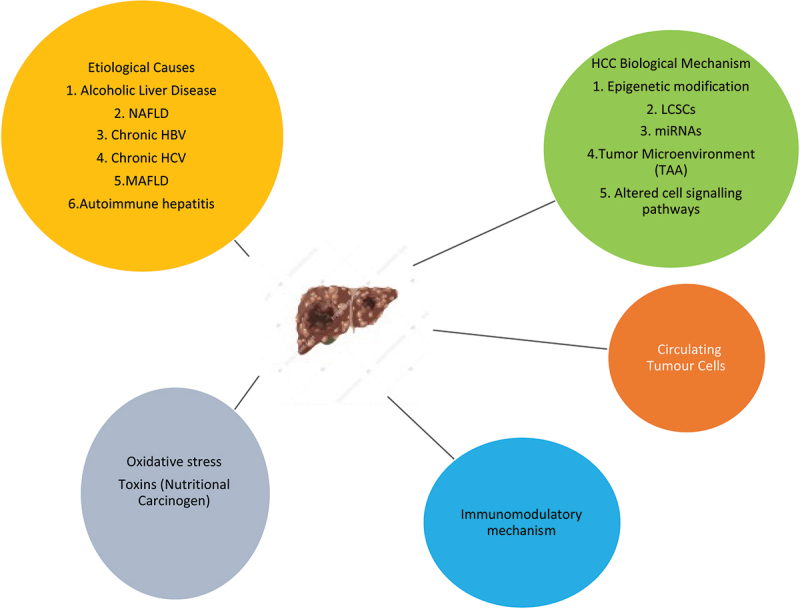
Summary of HCC progression mechanism.

Hepatocellular carcinoma (HCC) is the seventh most highly prevalent malignant tumor globally and the second most cause of mortality [1]. Early stages of HCC are characterized by dysplastic lesions frequently arising in chronic inflammatory liver disease or hepatitis that contribute to fibrosis and subsequently cirrhosis affecting liver function and often leading to death [2]. Globally, there are about 7.5 lakh new instances of HCC per year, ranking it the fifth most frequent cause of cancer in people. The mortality rate for HCC is exceedingly high; it is estimated that around 7 lakh people die from HCC each year, making it the third most prevalent cancer-related cause of death in humans [3]. With 3,95,000 cases per year and a prevalence of 35 per 1,00,000, China accounts for half of the global HCC burden [4]. According to currently available data in India, the age-adjusted incidence rate of HCC for men ranges from 0.7 to 7.5 and for women from 0.2 to 2.2 per 1,00,000 people per year. In India, there are 1.6% more HCC cases among cirrhotics per year. Around 90% of HCC cases develop in a background of cirrhosis but less than 5% of patients with cirrhosis progress to HCC per year [5]. However, the annual recurrence rate for HCC is 15%-20% which is higher than any other malignant neoplasm with high mortality [6]. In India, the male-to-female ratio for HCC is 4:1, with the age of the presentation ranging from 40 to 70. In India, the age-standardized death rate for HCC is 6.8/1,00,000 for men and 5.1/1,00,000 for women [7]. Each region has different HCC risk factors. The primary distribution of HBV and HCV infection has been linked to regional variations in the occurrence of HCC (Figure 1). In between 80% and 90% of HCC cases, one of two viruses is involved [8]. Multiple risk factors are known to cause HCC to develop at a younger age. It has been demonstrated that HBsAg-positive patients with HDV superinfection develop cirrhosis and HCC at a mean age of 48 years, as opposed to HBsAg carriers without HDV infection at a mean age of 62 years. HDV coinfection with HBV is related to accelerated liver damage [9]. In many reported studies, up to 30–40% of individuals with chronic liver disease or HCC there is no definite risk factor could be found. It has been suggested that nonalcoholic steatohepatitis (NASH), a more severe variant of nonalcoholic fatty liver disease (NAFLD), is the cause of cryptogenic cirrhosis. However, once cirrhosis and HCC are present, it is challenging to recognize the pathologic characteristics of NASH. In research evaluating the connection between the two diseases, obesity and diabetes, which are closely associated with NASH, have been indirectly linked to HCC [10]. Chronic alcoholic liver disease has emerged as one of the main HCC risk factors in the West. It is generally known that consuming a lot of alcohol (>50–70 g/day for numerous years) increases the risk of developing HCC. Patients with Child-Pugh Class A or B alcoholic cirrhosis had a yearly HCC risk of about 2.5% [11]. After quitting drinking, the risk of getting liver cancer decreases by 6 to 7% annually, and it also takes an estimated 23 years. There is proof that alcohol and HCV or HBV interact synergistically, likely leading to the promotion of cirrhosis [12]. Hepatocarcinogenesis has been demonstrated to be fueled by molecular changes at the genetic and epigenetic levels [13]. Copy number abnormalities (CNAs), somatic mutations at the genome level, gene expression at the transcriptome level and epigenetic modifications are some examples of the global molecular landscapes of HCC at many levels. Additionally, the capacity of next-generation sequencing (NGS) to unbiasedly capture all viral integration sites offers a whole new technique to comprehend the interaction between the host genome and the hepatitis B virus (HBV). The analysis of hepatocarcinogenesis at various molecular scales may provide novel therapeutic approaches. The creation of tailored therapeutics for this malignancy can be accomplished by the identification of important molecular processes and signaling pathways [14]. HCC develops with complex pathways that occur through the multi-stage biological process from normal hepatocyte to tumor transformation, including genetic and epigenetic modification, oxidative stress regulation, inflammation, and immunity participation [15]. Protein products of the biological process facilitate the identification and act as specific biomarkers for HCC [16]. Important risk factors include viral HBV and HCV, excessive alcohol, aflatoxin B intoxication, genetic disorders (hemochromatosis) and metabolic disorders (e.g. Diabetes mellitus (DM)) [17]. DM in Chronic Hepatitis B (CHB) persons has a high-risk factor for HCC [18]. Poor clinical outcome of HCC patients is majorly due to the resistance of HCC cells to the various treatments and tumor recurrence after curative therapies [19].

Liver cancer stem cells (LCSCs) play an important role in cancer initiation, metastasis, recurrence, and therapeutic resistance [20]. Chronic stressful events in liver progenitor cells give rise to inflammatory mediators such as TNF-α which forms the basis of cancer stem cells [21]. LCSCs are derived from the de-differentiation of mature hepatoblasts and biliary cells under the influence of genetic and epigenetic changes [22]. EpCAM, CD133, CD44, CD13, CD54, CD73, CD206, and CD34 are biomarkers for the identification of LCSC [23]. Shorter patient survival was linked to a considerably higher ratio of the insulin receptors A and B in human HCC that expressed stem/progenitor cell characteristics such as α-fetoprotein (AFP) and CK19 [24]. Current research indicates hepatic stem cells render a protective mechanism against genetic damage. Remarkably, stem cells are present in large numbers in the liver and have a capacity for a high rate of cell division during regeneration [25]. Therapeutic strategies involve trans-arterial chemoembolization (TACE), local radiofrequency ablation (RFA), and microwave ablation, mostly HCC cases that are diagnosed in advanced stages.

### Common mutations of protein-coding genes

The depletion of p53 a tumor suppressor protein leads to the dedifferentiation of mature hepatocytes into progenitor-like cells and further develops into HCC with a gene mutation in Wnt and Notch signaling pathways [26]. HCC is now broadly classified as proliferation and nonproliferation (inflammation type). Proliferative HCCs are typically more aggressive and less differentiated and are frequently associated with high serum AFP levels, TP53 mutations, and poor outcomes. Furthermore, proliferative HCCs may display TGFβ, MET, AKT, and IGF2 pathway activations. In contrast, the non-proliferative subgroup appears to be heterogeneous with less certain biological significance. Whereas a subset of non-proliferative HCC harbor mutation in β-catenin (encoded by catenin beta-1 (CTTNNB)) and show β - catenin pathway activation [27]. Mutant TP53 is probably related to HBV infection, while mutant CTNNB1 has been demonstrated to be the putative driver for alcoholic HCC [28]. Several analyses have further shown that they play a potential role in influencing numerous facets of the human cellular machinery e.g. in Deoxyribonucleic acid (DNA) repair and surveillance (TP53, CDKN2A, and RB1), Wnt/B catenin signaling (CTNNB1 and AXIN1), chromatin remodeling (ARID1A, ARID1B, and ARID2), oncogenic mitogen-activated protein kinase signaling (MAPK and RPS6KA3), oxidative stress (NFE2L2 and KEAP1), and histone modification (MLL, MLL3, and MLL4). There remains, however, a question concerning how these mutants function together. Inhibition of chaperone-mediated autophagy (CMA) limits tumor development, and the role of impaired CMA function is noted in neurodegeneration and cancer [29].

### Altered cell signaling pathways

Several studies have supported the fact that insulin-like growth factor (IGF) signaling pathway is involved in the occurrence of HCC [30]. A confined subset of cell-signaling pathways may be sequentially involved in hepatocarcinogenesis and tumor progression with metastasis, according to new evidence generated by using various model systems, even though our understanding of the altered signaling pathways in HCC is far from complete [31]. Growth factors and their accompanying receptors, such as the HGF receptor MET, IGF, and members of the ERBB family, are frequently dysregulated in HCC [32]. The IGF axis plays a critical role in promoting cell proliferation and preventing cell death. Allelic losses of IGF2R and overexpression of IGF2 are the most often documented aberrant characteristics in HCC, even in preneoplastic lesions. The Ras-MAPK pathway and the Phosphatidyl Inositol 3-Kinase pathway are both activated in response to an active IGF/IGF-1 R signaling pathway. These pathways stimulate a variety of biological processes, including protein synthesis, cell growth, differentiation, and survival. IGF-2 levels have been found to be higher in patients with HCC. IGF-2 may promote the growth of cancer cells in HCC patients because it binds to IGF-1 R as well [33].

The HGF/MET axis contributes to cell migration and proliferation. It plays a significant part in the renewal of physiologic life. 20–40% of HCCs have been reported to have MET activation, mostly due to overexpression of the receptor and its ligand. HGF/c-Met signaling pathways are deregulated in human cancer by overexpression of HGF or c-Met, gene amplification, mutational activation of c-Met, downregulation of Met-targeted miRNA, binding to other ligands, autocrine signaling or overly high levels of HGF [34].

**Figure 2.**
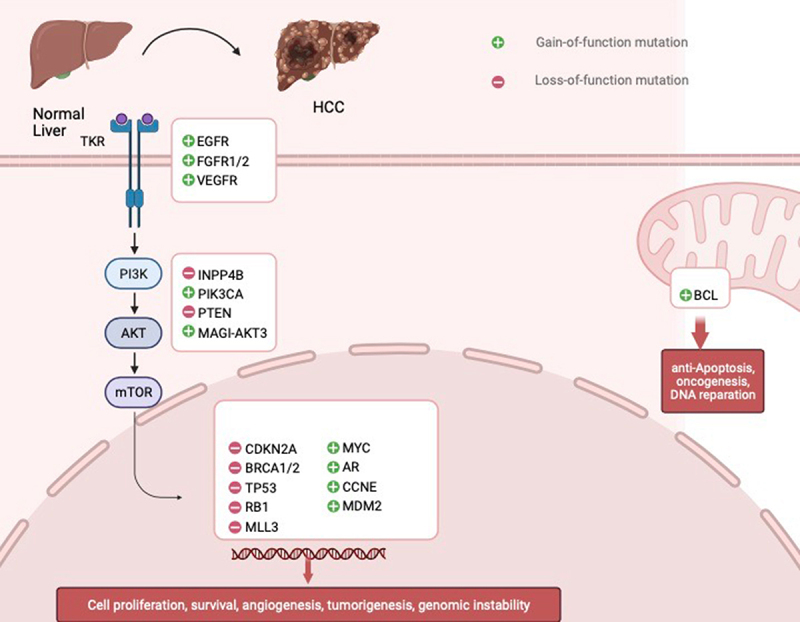
Altered cell signaling pathways in HCC.

Alterations in Wnt/-β-catenin, Notch, and Hedgehog signaling pathways (differentiation and development) lead to uncontrolled cancer cell proliferation in HCC. Mutations in the p53/p21 tumor suppressor gene cause cancer cells to multiply, which aids in the development, progression, aggressiveness, and metastasis of tumors. Changes in the signaling pathways that control growth factor receptors (such as VEGFR, FGFR, TGFA, EGFR, and IGFR) (Figure 2) or their cytoplasmic intermediates (such as PI3K-AKT-mTOR, RAF/ERK/MAPK) as well as important pathways for cell differentiation (such as Wnt/-catenin, JAK/STAT, Hippo, Hedgehog, and Notch). The NFE2L2/KEAP1 signaling pathway gene mutation facilitates the release of NRF2 from KEAP into the nucleus, which causes oxidative stress and the development of cancer cells. HCC is caused by the loss of the ARID1A/ARID1B/ARID2 complex, which mediates chromatin regulation and MLL signaling pathways. Cytokine and growth factor signaling are mediated by the JAK/STAT signaling network, as well as others, and all play crucial roles in controlling a wide range of essential biological processes [35].

Growth factors and their receptors predict the progression of HCC. Chronic insults from viral agents, metabolic syndrome, alcohol, and toxins cause uncontrolled cell proliferation, survival, angiogenesis, tumorigenesis, and genetic instability.

### The potential target of HCC and biomarkers

Although blood-based biomarkers AFP, AFP-L3, and DCP are available, early diagnosis of HCC is relatively low. Hepatic progenitor cells and cholangiocytes have the tumor markers cytokeratin 7 (CK7) and cytokeratin 19 (CK19), whereas normal hepatocytes do not. Oval cell marker (OV6) is involved in the development of putative progenitor cells. OV6-positive HCC cells have much higher levels of tumorigenicity, differentiation, and self-renewal than OV6-negative cells [36]. CD13 is a myeloid bone marrow-derived marker and CD44 antigen is a cell surface glycoprotein involved in cell–cell interactions, cell adhesion and migration, both of which are overexpressed in metastasis and have poor prognosis in HCC. Studies indicate that administering CD44-targeted DOX liposomes intravenously along with conventional HCC therapy substantially enhances tumor response [37].

Keratin 19 has a role in cancer cell proliferation and angiogenesis and its inhibition in HCC results in the control of cancer cell growth. CD133 is a pentaspan membrane glycoprotein that is a stem cell biomarker. In HCC patients, increased CD133 expression is discovered to be a standalone prognostic predictor for survival and tumor recurrence. Glypican 3 (GPC3) cell surface heparan sulfate proteoglycan is involved in cell signaling pathways via glycosyl phosphatidylinositol linkage. Normal hepatocytes do not have GPC3, whereas HCC cells display it on their surface. Previous studies have hypothesized that GPC3 upregulates TGF-β and c-Myc expression via triggering the traditional Wnt signaling pathway. Human antibodies HN3 and MDX-1414, as well as the mouse antibody YP7 that has been humanized, are utilized in HCC treatment to target GPC3. Asiaglycoprotein receptor (ASGPR) is a c-type lectin receptor primarily expressed on the sinusoidal surface of the hepatocytes which is over-expressed in early and advanced stages of HCC. We may expect to learn more in the near future regarding the function of ASGPR.

Transferrin receptor (TfR) (CD71) utilizes heme albumin as cargo to transport iron into human cells and is involved in the proliferation of cells. TfRs have a variety of advantageous traits that are employed in conjunction with cytotoxic drugs to destroy cancer cells. In cancer therapies, TfR antibodies are combined with chemotherapeutic drugs. TfR was created using self-emulsifying nanoparticles for the co-drug delivery of doxorubicin and cisplatin for HCC treatment. Folic Acid Receptor (FAR) membrane-bound surface protein binds to folates and folate conjugates with high affinity and is over-expressed in HCC. In vitro studies micelles created by folic acid that is loaded with doxorubicin and superparamagnetic iron oxide are administered to human HCC cell lines that overexpress the folic acid receptor. The somatostatin receptor (SSTR) is a receptor for ligand somatostatin, expressed abnormally in the HCC and may be key in the therapeutic targets for HCC treatments [38].

**Figure 3.**
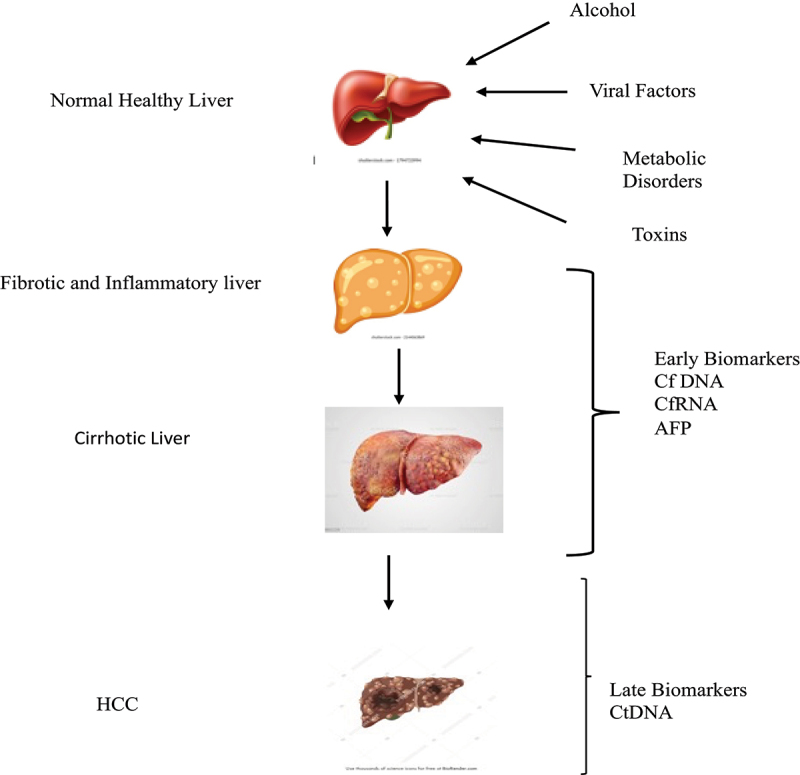
HCC development and biomarker. There is an importance of biomarkers in the early diagnosis of HCC.

Five metabolite panels that include methionine, proline, ornithine, pimethylcarnitine, and octanoyl carnitine were constructed as an early diagnostic tool for HCC. Circulating tumor cells (CTC) and circulating DNA (ctDNA) that derive from the tumor cells are being developed for the early diagnosis of HCC [39]. CTC is a group of crowded and scattered cells that are released into the circulating system from the primary tumors [40]. Cancer-like traits in cell-free DNA (cfDNA) indicate that cancer cells may release DNA into peripheral circulation. cfDNA produced by cancer cells and discharged into the bloodstream is now known as circulating tumor DNA. Despite certain difficulties in their identification, measurement, and characterization, CTCs provide important diagnostic and therapeutic information on HCC. To increase the effectiveness of clinical CTC tests, alternative liquid biopsies such as ctDNA and exosomes should be used in conjunction with CTC [41] (Figure 3).

### Role of miRnas in HCC

Overwhelming evidence has manifested the roles of nc RNA (non-coding RNA) in diagnosing and managing HCC. Growing attention has been paid to micro-RNAs (miRNAs) in the diagnosis and management of HCC. miR-125b [42], miR-122 [43], miR-21 [44], miR-22 [45], and miR-3197 [46] have been identified to be useful in the diagnosis of HCC [47]. Extracellular vesicles loaded with anti-tumor miR-31 and miR-451a were found to accelerate the apoptosis of HCCs, which may be helpful in the control of the development of the HCC [48].

### Epigenetic alterations

DNA is methylated by DNA methyltransferase (DNMT), an enzyme whose overexpression is associated with poor survival in HCC [49]. Regions that are usually affected by aberrant hypermethylation are promoter sequences of genes responsible for cell cycle regulation, apoptosis, DNA repair, metabolism of carcinogens and angiogenesis. Contrary to low methylation levels in the certain promoter region, these are also linked to the advanced histopathological grade of HCC [50]. Hypomethylation has been shown to activate proto-oncogenes including c-Jun and c-myc in HCC. Over the years, a number of epidrugs have been created. For the treatment of hematological malignancies, the majority of them are either in use or undergoing clinical studies. However, these substances are now being explored both experimentally and clinically in solid tumors, such as HCC. DNMTi inhibitors and acetone deacetylase inhibitors (HDACi) were the first epidrugs that the FDA authorized.

### Immunotherapies in HCC

Targeting HCC with nanoparticles and immunotherapy is a recent advancement in the treatment scenario. Tumor immunotherapies, including immune checkpoint inhibitors (ICIs), chimeric antigen receptor T cells (CAR-T), and bispecific antibodies (BsAb) have significant potential in cancer therapy [51]. BiTE is a type of BsAb that binds to CD3 and TAA and can entice T lymphocytes to attack cancer cells. By simultaneously releasing cytokines and lysing HCC cells, BiTE specifically killed the cancer cells. In a small number of patients, immune treatment with checkpoint inhibitors has demonstrated a potent anti-tumor effect, and the combination of the anti-PDL1 antibody atezolizumab and the VEGF-neutralizing antibody bevacizumab is currently the gold standard of care as the first-line therapy for HCC or will be in the near future. Whereas the anti-PD1 agents nivolumab and pembrolizumab are employed after TKIs in multiple regions. Checkpoint inhibitors used in immunotherapy have demonstrated anti-tumor potential in a group of patients [52].

Contrary to monoclonal antibodies, bispecific antibodies (BsAbs) are produced by largely utilizing recombinant DNA technology and have the ability to accurately and simultaneously bind two antigens or epitopes. BsAb can target immunological checkpoints and tumor-associated antigens (TAAs) to reverse immunosuppression in the tumor environment. It can also directly increase the activity of immune cells against tumors. As a result, they outperform monoclonal antibodies in terms of synergistic effects and have the ability to mediate a wide range of particular biological effects.

Recently, Adoptive Cell Therapy (ACT), an immunotherapy that fights cancer using the patient’s immune system or the immune system of a healthy donor, has become a crucial component of the cancer treatment process. ACT is a highly customized cancer treatment when compared to antibodies or other targeted drugs [53]. A diverse population of effector CD3+CD56+ natural killer T cells known as cytokine-induced killer (CIK) cells may be readily grown in vitro from peripheral blood mononuclear cells. Cytokine-induced killer cells are a heterogeneous cell population that can be expanded *in vitro* from PBMC with the addition of IFN-γ, anti-CD3 antibody, and IL-2 [54]. In fact, CIK anticancer activity has been enhanced by the creation of several new cytokines. By reducing the production of regulatory T (Treg) cells, which are known to decrease antitumor immunity, these cytokines can enhance cell proliferation and cytotoxicity [54].

Tumor Infiltrating Lymphocyte (TIL) is one of the key elements of the host immune system’s anti-tumor defenses, along with regulatory T cells (Treg), NK cells, T cells, and B cells. TILs, which are isolated from surgical cancer tissues and have a broad antigen recognition capacity, have a greater tumor-inhibitory effect than therapies that concentrate on a single antigen or mutation [55]. TILs can be expanded *in vitro*; however, it is challenging to separate them from the tumor tissues of HCC patients. Additionally, only a small number of HCC patients can tolerate lymphocyte elimination, which is necessary prior to TIL infusion [56]. Chimeric antigen receptor T cell (CAR-T), a novel cancer immunotherapy in which T cells are genetically modified to recognize specific TAA, is the main research focus of ACT. It is effective in treating hematological diseases [57]. CAR-T treatment for liver cancer is still under development because of the variety of solid tumors, the lack of precise targets, and the vulnerability to the tumor microenvironment [58].

The liver has a larger concentration of chimeric antigen receptor-natural killer cells (CAR-NK) than the spleen or peripheral blood. As a result, NK cells are thought to be crucial in the prevention of HCC and are a possible source for cell therapy in the treatment of HCC [59]. The method used to create CAR T cells may also be used to create CAR NK cells [60]. Due to their shorter lifetime than CAR T cells, CAR NK cells have an advantage in that they potentially lower the likelihood of an autoimmune reaction and tumor transformation. GPC3-specific CAR NK cells have been shown to produce cytokines and cause cytotoxicity *in vitro* when cocultured with GPC3+HCC cells [61]. T-Cell Receptor-Engineered T cell (TCR-T) is developed to selectively recognize the tumor antigen peptide-MHC complex, where T cells are modified with the exogenous TCR gene to create TCR-T cells. TCR-T cells that target HBV and HCV have been created and are now being tested for effectiveness and safety [62].

## Autoimmune hepatitis related HCC

Autoimmune hepatitis is a fairly uncommon disease that results from the progressive destruction of the hepatitis parenchyma through a loss of immune tolerance toward hepatocytes [63]. In a 4:1 ratio, females are afflicted more frequently than males. The range of AIH prevalence rates per 100,000 people is 4.8 to 42.9. In Asia, where the disease is more common and fatalities are higher. Although we have a limited understanding of the underlying causes of inflammation, mixed environmental, genetic, and epigenetic factors are all thought to be significant. For instance, significant links between non-HLA genetic variants and disease risk have been discovered, as well as human leucocyte antigen (HLA) relationships. Some are common but without a clear coding impact, such as in the gene locus for SH2B3, while others are uncommon yet functional, like in the genes AIRE, GATA-2, and CTLA-4. Two types of autoimmune hepatitis have been identified based on the presence of circulating autoantibodies in patient sera. Anti-nuclear antibodies (ANA), anti-smooth muscle antibodies (SMA), and anti-soluble liver antigen (SLA) are the hallmarks of type 1 AIH. Anti-liver-kidney microsome antibodies (LKM1) and/or anti-liver cytosol 1 antibodies (LC1) are markers for type 2 AIH patients. According to studies, gene polymorphism plays a significant role in AIH. Polymorphisms in the FAS gene promotor (position 670), vitamin D receptor, TNFA2 (TNF) gene (position 308) and CTLA4 (T-lymphocyte antigen-4) have all been connected to type 1 AIH. In autoimmune hepatitis (AIH) patients, liver histology shows portal and periportal inflammations. AIH does not follow the Mendelian pattern of inheritance, and no genetic locus has been identified in disease causation. Polymorphism in the human leukocyte antigen (HLA) locus on chromosome 6p21.3 is very common in AIH [64]. AIH causes cirrhosis and eventually HCC. Further research is required to survey the occurrence of HCC in AIH patients. CD4+ and CD25+ Tregs-mediated immune responses play an important role in the development of AIH-mediated HCC [65]. Mercaptopurine (MP) has recently been suggested as an alternate treatment for azathioprine intolerance; however, there is currently insufficient proof of its effectiveness in patients who do not react. Etiological causes and background of HCC progression are briefly shown in Table 1.

**Table 1.** Etiological causes and background of HCC development.

Etiological Causes	Background of HCC Development
Metabolic Syndrome	Chronic InflammationFat accumulation in Hepatocytes Insulin ResistanceDiabetes MellitusObesityHypertension Fat accumulation in Hepatocytes Insulin Resistance Diabetes Mellitus Obesity Hypertension
NAFLD	TNF αIL6LeptinResistin IL6 Leptin Resistin
HBV	DNA integrationGenomic InstabilityChronic InflammationTissue DamageEpigenetic AlterationRegeneration, Scarring and Stellate Cell ActivationFibrosis Genomic Instability Chronic Inflammation Tissue Damage Epigenetic Alteration Regeneration, Scarring and Stellate Cell Activation Fibrosis
HCV	Chronic inflammationNecrosisTissue damageEpigenetic AlterationRegeneration, Scarring and Stellate Cell ActivationFibrosis [66] Necrosis Tissue damage Epigenetic Alteration Regeneration, Scarring and Stellate Cell Activation Fibrosis [66]
Autoimmune disease	Epigenetic Variants Predisposition HLAD AllelesRegulatory mRNAs ExpressionsRisk Allele SH2B3CTLA4 VariantsFAS/FASL mutationsFOXp3 generationsAIRE mutationsGATA2 dysfunctionAltered immune mechanism Impaired CD4+ cellsCD8 + cytotoxic damageActivation of NK cellsB cell DifferentiationAutoantibodiesComplement activationSecreted cytokinesInterferon cell induced liver damageDefectous T regIL signaling in liver damage [67] Predisposition HLAD Alleles Regulatory mRNAs Expressions Risk Allele SH2B3 CTLA4 Variants FAS/FASL mutations FOXp3 generations AIRE mutations GATA2 dysfunction **Altered immune mechanism** Impaired CD4+ cells CD8 + cytotoxic damage Activation of NK cells B cell Differentiation Autoantibodies Complement activation Secreted cytokines Interferon cell induced liver damage Defectous T reg IL signaling in liver damage [67]
Nutritional carcinogen	Aflatoxin B1Chronic injuryGenetic and epigenetic alteration [68] Chronic injury Genetic and epigenetic alteration [68]
Alcoholic Liver Disease	NecrosisInflammationRegeneration, Scarring and StellateCell ActivationEpigenetic AlterationFibrosisGenetic Features TERT promoter MutationCTNNB1 MutationSignalling Pathways IL6-JAK STAT (more in steatotic Type) Wnt Beta catenin Signalling (more Cholestatic type)Chromosome Stability Chromosome 7 amplificationSteatotic type CRP+ Cholestatic TypeNuclear beta CateninCluster B/S3/iCluster 2G4 glass- InterferonPoly 7 subtypes WNT/Beta Catenine, CTTNNB1 Type -G5 G6 ClassIL6 -JAK STAT (more in Steatotic type) Wnt-Beta Catetine signaling (more in cholestatic type) Inflammation Regeneration, Scarring and Stellate Cell Activation Epigenetic Alteration Fibrosis **Genetic Features** TERT promoter Mutation CTNNB1 Mutation **Signalling Pathways** IL6-JAK STAT (more in steatotic Type) Wnt Beta catenin Signalling (more Cholestatic type) **Chromosome Stability** Chromosome 7 amplification **Steatotic type** CRP+ Cholestatic Type Nuclear beta Catenin Cluster B/S3/iCluster 2 G4 glass- Interferon Poly 7 subtypes WNT/Beta Catenine, CTTNNB1 Type -G5 G6 Class IL6 -JAK STAT (more in Steatotic type) Wnt-Beta Catetine signaling (more in cholestatic type)
Age	Up to 7-fold increased risk of HCC > 55Genetic Predisposition [69] Genetic Predisposition [69]
Overweight and Obesity	8-fold increase risk of HCC

## HBV related HCC

### Prevalence of HBV

Hepatitis B affects more than 296 million people globally, with a prevalence of 3.8%, a death rate of 820,000 cases, an incidence of 1.5 million people newly infected and 8.3 million individuals receiving treatment [70]. CHB virus infection is a predominant risk factor for HCC, with or without a high lining of liver cirrhosis, due to various mechanisms to promote hepatocarcinogenesis. HBV DNA is usually incorporated into the human genome and forms covalently closed circular DNA [71]. On molecular aspects, the HBV virus persistently remains inside the nucleus and acts as a template for viral replication [72]. Chronic HBV infection is the leading cause in approximately 50% of the cases of HCC, whereas currently, nonalcoholic steatohepatitis (NASH) is rapidly becoming a growing etiological concern.

### HBV genome and HCC

HBV DNA can be randomly integrated into the host cell genome. Although integrated HBV sequences cannot sustain viral replication, they can generate viral proteins, namely hepatitis B surface antigen (HBsAg) and transcriptional regulator HBx protein [73]. HBx is the key regulatory non-structural protein of the virus, related to the host cell cycle regulation, integration of HBV infection and carcinogenesis. HBx truncation can activate FXR signaling (bile acid receptor) leading to HCC [74]. HBx localizes its effect through signal transduction in the cytoplasm, transcription in the nucleus and mitochondrial activity in the mitochondria. There are four primary processes that support the development of HCC as a result of the transactivation of viral and cellular genes [1]: integration of the HBx gene into the hepatocyte genome, which encourages genetic instability [2]; cause of oxidative stress through the interaction with the mitochondrial as well as other cellular proteins [3]; activation of cell survival signaling pathways and inactivation of tumor-suppressors; and [4] induction of acetylation of histone, methylation, and microRNA expression (epigenetic modification) [75]. ASPP1 and ASPP2 gene expressions are reduced by HBx by DNA methylation modification, which prevents association with the p53 a tumor suppressor protein, which inhibits HCC apoptosis, promotes HCC growth and is closely related to the occurrence of early liver cancer [76]. C- terminal truncated HBx promotes HCC by induction of CD133+ LCSCs and its tumor-initiating capacity by regulating FXR pathway and drug metabolism [77]. Therefore, HBx has a tendency to affect a variety of proto-oncogenic signaling pathways implicated in inflammation and proliferation, including the NFk-β, JAK-STAT, protein kinase C, Src, Survivin, and PI3K cascades [78]. The breakdown of the fibrous capsules of the tumors by matrix metalloproteinases (MMP) is how HBx increases epithelial–mesenchymal transition (EMT) and metastasis. The direct transcription product of cccDNA, HBV-pg RNA (Pregenomic RNA), encourages the development of HBV-related HCC [79,80].

### HBV induced epigenetic alterations and HCC

HBV affects m^6^A modification of several host RNAs, including phosphatase-tensing homolog (PTEN), a known tumor suppressor. HBV affects innate immunity by inhibiting IRF-3 nuclear import and development of HCC by activating the PI3K/AKT, p38MAPK, JNK, ERK, NF-kß, WNT ß catenin, hypoxia-inducible factor 1α, Notch, and Hodge pathways [81,82]. PIVKA II des-gamma carboxyprothrombin an abnormal form of the coagulation protein prothrombin and alpha-fetoprotein (AFP) are both useful biomarkers for HCC [83]. In recent years, microRNAs have been recognized in the diagnosis and prognosis of HCCs and influence the biological behavior of HBV-related HCC [84]. Fibrotic degeneration and progression of HCC in chronic viral hepatitis are related to the activity of immune response in the liver. In HBV infection, the accumulated CD4+ and CD8+ are incapable of clearing this virus [85]. Some studies have reported the association between mutations in the HBV preS region and HCC [86]. Gao Q et al. study significantly five mutated genes were identified, namely TP53 (58%), CTNNB1 (19%), AXIN1 (18%), KEAP1 (7%), and RB1 (6%). Moreover, other genes such as TERT and RPS6KA3 hold repeatedly somatic mutations in HBV-associated HCC. Also, even if HBV-associated HCCs influence the latter mutations, they are more significant in HCV-associated HCC (TERT), 60–80% in HCV-HCC vs. 30–40% in HBV-HCC; ARID2, 18% in HCV-HCC vs. 2% in HBV-HCC [87]. The deletion mutation in the pre-S region of HBV correlates with the virus escaping from the host immune attack [88]. CK19 belonging to the family of keratins plays a key role in tumor malignancy through overexpression and can be used as a potential biomarker to predict adverse prognosis after surgery and adjuvant therapy in HBV-related HCC patients [89]. HBV-related HCC is characterized by the upregulation of genes associated with cell cycle control and monocytes/macrophage activation and the downregulation of genes involved in various cell metabolism [90]. Tregs play a suppressive role in HBV-related HCC by interacting with other cells or by secreting cytokines such IL-2, IL-10, TGF-β and IL-35 [91]. HBV induces PD-1 overexpression in Tregs which leads to a higher proliferation of HCC tumorigenesis [92]. The higher PD-1 levels in peripheral blood were associated with a higher rate of tumor recurrence and progression [93]. HBV suppresses the activation of CD8^+^ T cells via granzyme A/B and perforin. Higher accumulation of Tregs and reduced infiltration of CD8^+^ T cells were observed at the tumor site of liver [94]. Patients with chronic HBV infection had higher expression of the inhibitory receptors NKG2A, TIM-3, and PD-1, and their ability to secrete IFN-γ and TNF-α was lower, which was related to the development of HCC [95]. Moreover, the standard treatment of HBV-related HCC is currently unavailable. Blockade of T cell immunoglobulin and ITIM domain (TIGIT) alone enhanced the antitumor activity of CD8^+^ T cells during the progression of HBV-related HCCs in a spontaneous HCC mouse model [96]. ETV, TDF, and TAF have potent antiviral activity and a high barrier to resistance [97]. However, the effects of entecavir (ETV), tenofovir disoproxil fumarate (TDF), and tenofovir alafenamide (TAF) in the treatment of chronic hepatitis B (CHB) and its development/suppression of hepatocellular carcinoma (HCC) remain unclear. These contentious problems must be resolved and a consensus reached by additional clinical research or trials involving a larger patient population and longer follow-up.

## NAFLD related HCC

### Prevalence of NAFLD

It is estimated that 32.4% of the people globally have NAFLD [98]. NAFLD includes nonalcoholic steatohepatitis, which is characterized by inflammation and hepatocyte ballooning with or without fibrosis, as well as simple hepatic steatosis [99]. There was 5.4-fold increased risk of HCC in the non-cirrhotic liver for NAFLD-related HCC and 5-fold for metabolic syndrome (MetS) related HCC [100]. Men are more prone than women to acquire NAFLD, and the sixth decade of life is when the incidence is at its highest [101]. NASH is the second most common reason for HCC liver transplants, and it increases the chance of developing HCC compared to those with other liver conditions [102,103]. Despite the fact that cirrhosis is the main risk factor for the development of HCC, a sizable part of HCC linked to NASH arises in livers with or without liver fibrosis [104].

### Impact of insulin resistance and type 2 diabetes mellitus in NAFLD and HCC

Insulin resistance (IR) may be one of the most prevalent underlying risk factors for T2DM, which increases the probability of developing HCC by two to three times compared to other metabolic diseases [105]. IR promotes lipolysis and the release of adipokines that lead to ER stress, ROS-mediated oxidative stress, and mitochondrial dysfunction. Additionally, it causes adipocyte dysfunction and increased adipose tissue development. The three main causes of the development of NAFLD-related HCC are lipid buildup, lipotoxicity, and insulin resistance [106]. The complex interplay between environmental, genetic, and metabolic factors results in an imbalance between intrahepatic lipid retention and disposal. The relationship between NAFLD and metabolic dysfunction has recently led to the new term ‘MAFLD’ (metabolic-associated fatty liver disease), which underlies the coexistence of hepatic steatosis and metabolic syndrome [107].

### NAFLD-induced epigenetic alteration and HCC

The development of HCC in NAFLD may also be influenced by genetic variation. Single nucleotide polymorphisms (SNPs) in the immunoregulatory genes (MICArs2596542, CD44rs187115, PDCD1rs 7421861, and rs10204525), superoxide dismutase 2 (SOD2) and patatin-like phospholipase domain containing 3 (PNPLA3) [108,109] may be involved in the development of the HCC [110]. Due to its impact on the activity of retinyl-palmitate lipase, PNPLA3 appears to prevent hepatic stellate cells from producing and releasing retinol [111]. Transmembrane 6 superfamily member 2 (TM6SF2) gene is responsible for regulating lipid metabolism in the liver. Polymorphism of this gene is associated with elevated liver fat levels and advanced liver fibrosis. Membrane-bound O-acyltransferase domain containing seven transmembrane domains (MBOAT7) are integral transmembrane enzymes that are found in all kingdoms of life. Acyl-coenzyme A: cholesterol acyltransferase and diacylglycerol acyltransferase 1 are responsible for lipid biosynthesis or phospholipid remodeling. Mutation in this gene causes lipid accumulation in the liver cells with altered metabolism, causing hepatocyte ballooning and leading to steatohepatitis. Glucokinase regulator (GCKR) is a protein that inhibits glucokinase in the liver and pancreatic islet cells’ rare non-sense mutation in the GCKR gene that causes the progression of NAFLD [112]. The GCKR mutation that led to the P446L protein variant appears to be the causative variant that underlies the link with hepatic fat accumulation.

### NAFLD induced alterations in cell signaling pathways and HCC

In IR, obesity, metabolic syndrome, and NAFLD-related HCC, PI3K-AKT-PTEN, osteopontin (SPP1), and CXCL 10 [113] expression are frequently amplified [114]. Adiponectin, which is secreted by adipose cells in obesity, increases insulin resistance, plasma triglycerides, LDL cholesterol, the amount of fat in the liver and which leads to the likelihood of developing NASH and NAFLD [115].

Autophagy, a self-cannibalistic process, in which the cell digests its own contents, is a major pathway for lipid catabolism. Lipid accumulation, ER stress, and deregulated cytokines expression contribute to autophagy deficiency [116]. Obesity also increases the risk of HCC in populations already at a high risk of HCC, such as those with cirrhosis [117]. Hepatokines and adipokines are organ-specific cytokines released by the liver, which are important in metabolic homeostasis of the liver, which is altered in the NAFLD-related HCC, and both will be potential markers of disease progression [118,119]. Increased iron absorption and deposition are observed in NASH patients, and it has also been related to the development of HCC and the underlying mechanism might be related to oxidative DNA damage [120].

### Role of miRnas and exploring biomarkers in NAFLD-related HCC

Non-coding miRNAs, miR-122, miR-192, and miRNA 194 are all reportedly decreased in both NAFLD and HCC [121]. Some miRNAs is reportedly elevated in the NAFLD-related HCC. miRNAs alter hepatic lipid metabolism and enhance free fatty acid oxidation, contributing to NAFLD progression. miRNAs also interfere with the c-myc and epithelial–mesenchymal transition (EMT) pathways that promote HCC [122]. Serum biomarkers are crucial in the diagnosis of HCC before clinical symptoms appear, and miRNAs are crucial therapeutic targets [123]. TNF-α an inflammatory cytokine released by macrophages and monocytes during acute inflammation and IL6 pro-inflammatory cytokine expression were shown to be elevated in NAFLD-related HCC [124]. miR-223, a miRNA with anti-inflammatory functions, was reported to ameliorate hepatic inflammation in NASH via targeting C×c110 interferon-gamma-induced protein 10. It is secreted by several cell types in response to INF **γ** and transcriptional coactivator with PDZ-binding motif (TAZ) in hepatocytes [125]. Studies have shown that overt NAFLD causes the loss of mitochondrial flexibility and mitochondrial dysfunction in hepatocytes. Additionally, these signals (mito DAMPs) cause inflammation and fibrosis, creating an unfavorable microenvironment in which some hepatocytes choose anti-apoptotic programs and mutations that may promote survival and growth.

## HCV related HCC

### Global prevalence of HCV

Hepatitis C affects more than 56 million people globally, with a prevalence of 0.8%, a death rate of 290,000 persons, and 9.4 million people receiving treatment from 2015 to 2019 [70,126]. According to meta-analysis research, in patients with HCC associated with HCV who are not subjected to direct-acting antivirals (DAA), the recurrence rates of HCV infection at 6 months and 2 years were 7.4% and 47%, respectively [127]. DAA treatment was linked to a significantly lower chance of mortality [128].

### HCV-induced epigenetic alterations and HCC

Downregulation of immune genes, particularly T cell-related genes within the tumor, and overexpression of oxidative stress genes associated with necroinflammatory-mediated hepatocarcinogenesis outside the tumor are characteristics of HCV-related HCC. HCV core protein epigenetically affects hepatocytes apoptosis. In particular, the gamma Gly-Gly-Gly levels were substantially greater in HCV-associated HCC than in HBV-related HCC, while serum glutamic acid concentrations were significantly lower in HCV-related HCC patients than in HBV-related HCC [129]. Gene-specific hypermethylation or hypomethylation, global genomic hypomethylation, aberrant expression of DNA methyltransferases, histone-modifying enzymes, altered histone modification patterns, and aberrant expression of microRNAs are all characteristics of the altered epigenome in HCC. These changes can affect the expression of oncogenes, tumor suppressor genes, and other tumor-related genes, changing the cancer development pathways over time [130]. The well-known elevated expression levels of DNMT1 and DNMT3B associated with the HCV core have been linked to the alteration in host DNA methylation status, which was primarily found in HCV core-expressing cells.

### HCV-Induced epigenetic hallmark post cure of infection and DAA therapy

SVR, post-treatment blood albumin levels, and liver stiffness measurements are all linked to predicting the risk of HCC [131]. Surveillance of HCC is mandatory for patients who achieved SVR after DAA therapy [132]. While DAA therapy removes the virus from the cell, it is unable to reverse the concurrent epigenetic marks that have already been created and linked to the risk of HCC [133]. HCV protein NS5A is associated with the epithelial to mesenchymal transition (EMT) and somatic mutation in the telomerase reverse transcriptase promoter (TERT), which promotes fibrogenesis and tumor development and metastasis [134,135]. Due to HCV infection, tumor suppressor genes become hypermethylated and inactivated, disrupting the balance and causing malignant cancer hallmarks [136]. RAS protein activator like 1 (RASAL1), Egl-9 family hypoxia-inducible factor 3 (EGLN3), CUB sushi Multiple domains 1 (CSMD1), cyclin-dependent kinase inhibitor 2A (CDKN2A), BCL6 corepressor like 1 (BCORL1), zinc finger protein 382 (ZNF382) a member of zinc finger family, RUNX family (RUNX3), lysyl oxidase (LOX), RB transcriptional corepressor 1 (RB1), and tumor protein p73 homology to p53, all tumor suppressor genes are downregulated in HCV-related HCC [137]. The neuronal protein, synuclein gamma (SNCG), is highly expressed in advanced hepatocellular carcinomas and demethylation in tumor tissue of HBV and HCV-related HCC [138]. Autophagy favors HCV replication and inhibits cell apoptosis [139]. NS5A non-structural protein of HCV is shown to disrupt mitochondrial dynamics, thus increasing ROS production [140].

## Metabolic related syndrome (MAFLD)

Metabolic-associated liver disease (MAFLD) increases the risk of the development of HCC [141]. Despite the high prevalence of MAFLD and T2DM in India, there is no clear evidence or data on the prevalence of MAFLD-HCC among the general population. MAFLD independently increases the risk of HCC development by 7.3-fold in patients with CHB [142]. A recent estimate suggested that potential staggering 9,30,000 people in India might have MAFLD-HCC [143]. Control of obesity and diabetes could be beneficial in MAFLD-related HCC [141]. MAFLD is regarded as the main catalyst of HCC, and numerous studies have suggested that MAFLD could develop into HCC without progression through cirrhosis [144]. HCC is favorably correlated with metabolic illness, insulin resistance, and high BMI [142]. Strong correlations exist between the onset of MAFLD and the genetic variants Rs641738 and MBOAT7 [145]. MAFLD differs from other common causes of HCC, such as chronic viral hepatitis and heavy alcohol use, and there are no simple, highly effective therapies directed against MAFLD. Mostly unlike other cases, MAFLD-related HCC can arise in the absence of cirrhosis. Neutrophil infiltration is the potential hallmark of MAFLD [146]. Zhou et al. study showed that neutrophils could enhance the recruitment and polarization of macrophages and suppress regulatory T cells to accelerate HCC progression [147].

## Genetic Predisposition in HCC

Dysfunctional apolipoprotein B (ApoB 100) secreted from APOB genetic variants leading to a series of impairments of lipid export from hepatocytes within very low-density lipoproteins are responsible for the development of severe hepatic steatosis [148]. Somatic mutation in APOB in NAFLD-HCC frequently occurs but the mechanism behind it is not yet clear [149]. The gene Sequestosome −1 (SQSTM1) encodes for p62, a component of Mallory Denk Bodies and hyaline granules. SQSTM1 buildup is a sign of defective autophagy, which promotes carcinogenesis [150]. P62 is a classical receptor of autophagy involved in cell signaling and survival which accumulates in the cytoplasm of damaged liver cells in NASH and HCC [151]. HCC Hepatoblastoma has been shown to contain somatic mutations in the genes TP53, MET, CTNNB1, PIK3CA, AXIN1, and APC. HBV and HCV are major causes of viral hepatitis that lead to the development of cirrhosis and HCC. It involves the interaction of HBV DNA integration into host genetic machinery that may contribute to the malignant liver transformation of benign liver cells. HBV integration and viral particle replication lead to DNA hypermethylation and oxidative stress in mitochondria and affect normal liver function. Immunological and inflammatory responses induce the activation and overexpression of telomerase reverse transcriptase (TERT), a ribonucleoprotein polymerase that maintains telomere ends by adding the telomere repeat TTAGGG. Telomere length changes brought on by HBV integration with the TERT promotor sequence result in the development of HCC [152]. HBx and HBsAg gene integration into multiple domains of myeloid/lymphoid or mixed-lineage leukemia 4 (MLL4) leads to promoting the tumorigenicity of HCC via activation of the Wnt-β catenin signaling pathway. The retinoic acid receptor beta (RAR-β), a nuclear transcriptional regulator belonging to the thyroid hormone steroid receptor superfamily, binds to retinoic acid. It is a biologically active form of vitamin A which mediates cellular signaling, cell growth, and differentiation by activating cyclin-dependent kinase (E1/A2 (CCNE1/A2)) which is important in the cell cycle. RAR-β plays an important role in deactivating hepatic stellate cells. RAR- β expression is significantly reduced in cirrhosis and HCC [153]. The extracellular matrix protein FN1 ROCK1 is significantly expressed in HCC and is implicated in cell adhesion, proliferation, migration, and differentiation. It can be utilized as a biomarker for the early identification of HCC coupled with AFP [154]. Sentrin-specific peptidase 5 (SENP5), which is mostly found in the nucleolus and is greatly overexpressed in HCC, is involved in the release of mature ribosomes. According to research, SENP5 is necessary for HCC cell proliferation. It could be a promising therapeutic target for HCC in the future [155]. Angiopoietin 1 and angiopoietin-2 along with VEGF are overexpressed in HCC. Angiopoietins are involved in tumor angiogenesis in HCC [156]. TLR2/TLR9 are key innate immunity receptors participating in an immune response which is activated by tumorigenesis of the liver. Some studies have investigated and reported on the connection between TLR2 polymorphisms and the development of tumors [157]. Platelet-derived growth factor receptor α (PDGFRα) involves in the development of liver fibrosis and deposition of collagen in a pericellular and perivenular pattern, which is dysregulated and highly activated in HCC [93]. TP53 and CTNNB1 are the most prevalent mutations affecting 25% −30% of HCC patients [158].

Mutational Process in the liver carcinogenesis [159] AAV - (Adeno-associated virus), HCC - (Hepatocellular carcinoma)

Summary of the mutational process operating in hepatocellular carcinoma and chronic liver disease and their relationship to the specific risk factors and carcinogenesis Table 2.

**Table 2.** Genetic Predisposition of HCC.

Signatures of causes	Mutations	Types of mutation	References
Polycystic aromatic hydrocarbon	Tobacco-Related (substitution mutation)	C to A substitutions	Jessica L Petrick *et al.*, 2018
Liver-specific Alcohol Related	Alcohol-related (CTNNB1)	T to C in ATN trinucleotide	Sandra Rebouissou *et al*., 2016
HBV and AAV2	Insertional Mutagenesis	TERT, CCNE1, CCNA2	Cyriac A philps *et al*., 2021
Mismatch Repair Defect	DNA modifications	C to T and Deletion Mutation	Graeme A Macdonald *et al.*, 2003
Age	Gene Modification	Demethylation of Methylcytosine	Manar Atyah *et al.*, 2018
Aristolochic Acid	Substitution Mutation	T to A in CTG trinucleotide	Jean Charles and Eric Latouze 2019
Aflatoxin B1	Substitution Mutation	C to A in GCC trinucleotide,	Laura Gramantieri *et al*., 2022

## Alcoholic liver disease related HCC

Oxidative stress by reactive oxygen species (ROS) emerged from alcohol metabolism, increasing inflammation, and iron deposition, leading to higher chances of the occurrence of hepatocarcinogenesis [160]. ROS aggregation affects DNA structurally and functionally, leading to cell cycle arrest and apoptosis. Subsequent genetic modification such as dimorphism in the 2 G-MPO alleles in combination with the 2A SOD2 allele was found to be an independent risk factor for HCC [161]. The HFE gene encodes the human homeostatic iron regulator protein that regulates circulating iron uptake and interaction of transferrin receptors with transferrin. The HFE gene mutation leads to iron overload and the progression of HCC [162]. s Adenosyl L methionine (SAMe) maintains the methylation process of the genes in which alcohol interferes in the synthesis of SAMe leads to direct induction of HCC [163]. Excess fat accumulation in hepatocytes happens through the activation of sterol regulatory element-binding protein (SREBP-1) and downregulation of peroxisome proliferator-activated receptor-α (PPAR-α) affects the fatty acid beta-oxidation (FAO) [164]. Activation of stellate cells from dying hepatocytes transdifferentiates to myofibroblasts that secrete extracellular matrix (ECM) components collagen type I, III, and IV, fibronectin, hyaluronan, and proteoglycan [165]. Ballooned and dying hepatocytes induce hedgehog ligands that trigger hedgehog-responsive genes including the family zinc finger 2 (Gli2), alpha-smooth muscle actin (α-SMA), and vimentin that activates hepatic stellate cells. Variants of alcohol dehydrogenase (ADH1C), manganese superoxide dismutase (MnSOD), and glutathione S transferase (GSTM1) can be used to identify the early diagnosis and assist in the management of ALD [166].

## Future direction and research gap

The clear molecular mechanism behind the development of HCC is yet to be elucidated. Genetic predisposition and specific targets in gene therapy and immune-targeted therapy are the recent new developments in the medical management of HCC. Three areas will substantially benefit from this approach, prognosis assessment, prediction of treatment response, and identification of novel targets for molecular therapies. Vaccinations and antiviral therapies reduce the risk of HCC, meanwhile there is a steady increase in the occurrence of HCC. Awareness and strategic approaches are needed to reduce the risk of NASH and HCC. Serum biomarkers that replace the imaging and screening modality or even diagnosing modality in the future. Early diagnosis and medical management of HCC are more important in the future. The treatment plan for HCC is being developed and made more specific as the molecular features of HCC are understood. More investigation is required to comprehend the particular immunotherapy resistance mechanism and find more predictive biomarkers to aid therapeutic decision-making [167].

A combination of atezolizumab and bevacizumab in advanced HCC as a first-line treatment has been shown to be more successful than sorafenib. Alternatives for follow-up therapy to immunotherapy, Phase III clinical trials are being conducted in several settings. In immunotherapy, ICIs such as LAG3, TIM3, and GITR are steadily proving their efficacy in targeting PD-1/PDL1 and CTLA-4. Additional research is being done on ADCs, CAR-T, and BiTEs, among other immunotherapeutic modalities. The therapy of HCC with clinical medicines alone might not be enough.

LCSCs promote carcinogenesis by altering cell-signaling pathways and gene expression related to specific proteins involved in normal biological processes. The malignant characteristics of HCC, including plasticity, heterogeneity, EMT, angiogenesis, and vascular mimicry, are maintained and promoted by LCSCs. Future research will concentrate on increasing the precision of LCSCs isolation and patient-specific LCSCs identification. Research in LCSCs needs to elucidate and describe the molecular processes regulating LCSCs at different phases of tumor development. This enhances the efficiency of pharmacological treatment for treating HCC. We conclude that there is promise for a treatment for HCC by focusing on LCSCs and their biological characteristics.

**Figure 4.**
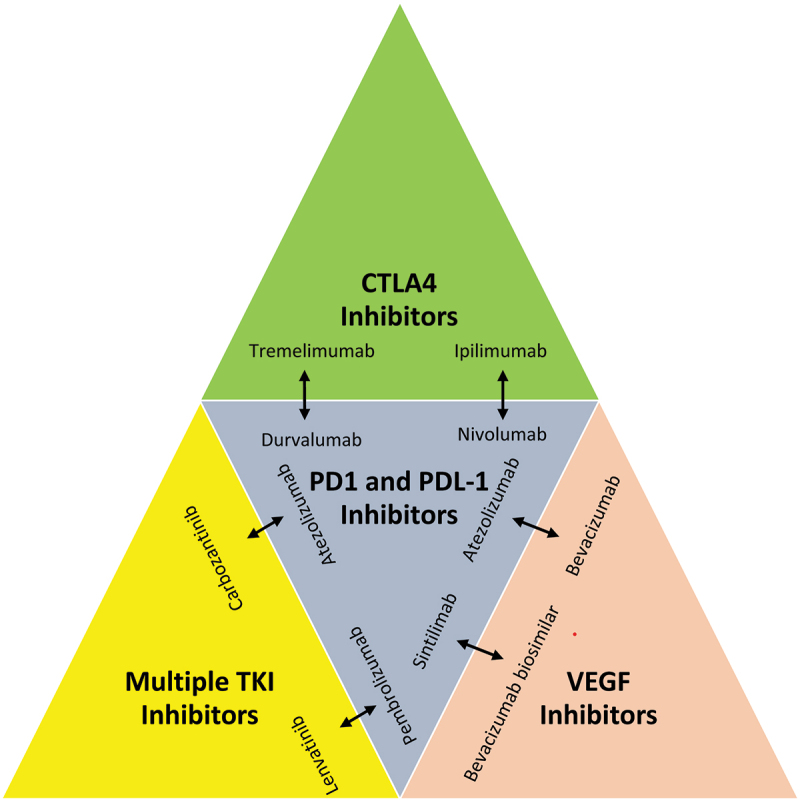
Combination of immunotherapies in HCC.

Recently, CAR-T therapy has received attention and is being researched for its potential therapeutic use in a variety of tumors, primarily hematopoietic tumors. Studies have shown that GPC3-targeted CAR-T inhibits the development of HCC *in vivo*. CAR-T, which is antigen-targeted for LCSCs, may be employed to directly eradicate LCSCs. Combinations of immunotherapies and ongoing clinical trials as a recent advancement in the strategic therapy in HCC (Figure 4). In a mouse model, BiTE demonstrated its efficacy in eradicating HCC tumors. Due to heterogeneity and complicated immunogenicity, antibodies that were effective in animals may not be effective in people. This is because various species have varying immune rejection responses and the human body may react differently to antibodies derived from animals. Their efficiency and security require improvement. The immunogenicity, poor stability, and high cost of the antibodies employed in immunotherapy may have restricted their clinical usage.

Multiple tyrosine kinase inhibitors, VEGF inhibitors, CTLA4 inhibitors, PD1 and PDL1 inhibitors are being tested in combination therapy. It demonstrates some promising results in the treatment of hepatocellular carcinoma.

Advancement of transcriptomics and metabolomics will help in early diagnosis and surveillance of HCC. HCC is caused by multiple risk factors involving various pathways. The combination of biomarkers that have been developed and optimized will be more useful for detecting HCC early and predicting its prognosis. The detection of cell-free DNAs in peripheral blood will aid in the early-stage diagnosis of HCC. In the later stages of HCC, circulating tumor DNA (CtDNA) is useful for evaluating tumor heterogeneity. There is a major gap in the determination of the early stages of HCC with specific biomarkers. It stresses the importance of including genomic analysis in the clinical practice protocol of HCC.

## Conclusion

Several medications are now being developed that target several molecules that are dysregulated in HCC and take the role of hepatocarcinogenesis. A greater and better understanding of the disease has resulted from the extensive quest for biomarkers or molecular signatures linked to the development of HCC. Using multi-omics technologies, researchers have thoroughly studied the immunological microenvironment, gut microbiota, liquid biopsy, and personalized medicine Numerous biomarkers have been developed in research into the molecular biology of hepatocarcinogenesis that would give additional information about HCC biological behavior mutations and recurrence to those derived from standard histological characteristics.

## Data Availability

Data sharing is not applicable to this article as no new data were created or analyzed in this study.

## Abbreviations

AAV: Adeno Associated Virus
ADH1C: alcohol dehydrogenase 1 C
AFP: Alpha Feto Protein
ANGPT1: Angiopoietin 1
ANA: Anti nuclear antibodies
APOB: Apolipoprotein B
ARID1A: AT rich interactive domain-containing protein 1 A
ARID1B: AT rich interactive domain-containing protein 1 B
ARID2: AT rich interactive domain-containing protein- 2
ASSP1: Argininosuccinate Synthesae 1 pseudogene 1
AXIN1: Axis Inhibition Protein 1
BCORL1: BCL6 Corepressor Like 1
CAR-T: chimeric antigen receptor T cells
CAR NK: Chimeric Antigen receptor T cell (CAR-T cell)
CCNE1: Cyclin E 1
CDKN2A: Cyclin dependent kinase inhibitor 2A
CK 19: Cytokeratin 19
CMA: Chaperone mediated Autophagy
CSMD1: CUB sushi Multiple domain 1
CTC: Circulation tumor cells
CTNNB-1: Catenin Beta 1
DNMT-1: DNA cytosine 5 methyltransferase 1
EGLN3: Egl-9 Family hypoxia-inducible factor 3
EGFR: Epidermal Growth Factor Receptor
EMT: Epithelial-mesenchymal transition
ETV: Entacavir
FN1: Fibronectin 1
GCKR: glucokinase regulator
GPC3: Glypican −3
GSTM1: glutathione S transferase
HCC: Hepatocellular Carcinoma
IGF2: Insulin like Growth Factor −2
IGF2R: Insulin like Growth Factor 2 Receptor
JNK: c-Jun terminal kinase
KEAP1: Kelch like ECH Associated protein −1
LC1: Liver cytosol 1
LOX: Lysyl Oxidase
LKM: Liver kidney microsome antibodies
MBOAT1: membrane-bound o-acyl transferase domain containing 1
MnSOD: Manganese superoxide dismutase
MET: Mesenchymal Epithelial Transition (HGF)
MLL4: Myeloid/lymphoid or Mixed-Lineage Leukemia 4
MMP: Matrix metalloproteinase
NFE2L2: Nuclear Factor (Erythroid-derived 2)like 2 (NRF2)
NKT: Natural Killer T cells
OV6: Oval cell marker
PDGF: Platelet Derived Growth Factor
PIK3CA: Phosphatidylinositol-4 5 Bisphosphate 3 Kinase
PIVKA II: protein induced by vitamin 2
RAR beta: Retinoic Acid Receptor Beta
RASAL 1: RAS protein activator like 1
ROCK1: Rho-associated coiled-coil containing protein −1
RUNX-3: Runt related Protein
RB1: Retinoblastoma associated protein −1
RPS6KA3: Ribosomal protein S6 kinase A3
SENP5: Sentrin-specific protease 5
SAMe: s adenosyl L methionine
SMA: Smooth muscle antibodies
SOD: Super Oxide Dismutase 1
SQSTM1: Sequestosome −1
SLA: soluble liver antigen
SMA: Smooth Muscle Antigen
SNCG: synuclein gamma
TACE: Trans Arterial Chemo-embolisation
TAA: Tumor-associated antigen
TAF: Tenofovir alafenamide
TCR-T: T cell Receptor -Engineered T cell
TERT: Telomerase Reverse Transcriptase
TNFA2: Tumor Necrosis Factor alpha 2
TIL: Tumor infiltration Lymphocyte
TLR2/TLR9: Toll-like receptor 2/Toll like receptor 9
TP 53: Tumor Protein 53
TM6SF2: Transmembrane 6 superfamily member 2
TM6SF2: Transmembrane 6 superfamily membrane 2
VEGF: Vascular endothelial growth factor
ZNF 382: Zinc Finger Protein 382

## References

[1] McGlynn KA, Petrick JL, El-Serag HB. Epidemiology of hepatocellular carcinoma. Hepatology. 2021;73(S1):4–13. doi: 10.1002/hep.31288 PMC7577946 32319693

[2] West J, Card TR, Aithal GP, et al. Risk of hepatocellular carcinoma among individuals with different aetiologies of cirrhosis: a population-based cohort study. Aliment Pharmacol Ther. 2017;45(7):983–990. doi: 10.1111/apt.13961 28144999

[3] EASL–EORTC clinical practice guidelines: management of hepatocellular carcinoma. J Hepatol. 2012 Apr;56(4):908–943. doi: 10.1016/j.jhep.2011.12.001 22424438

[4] Ozakyol A. Global Epidemiology of hepatocellular carcinoma (HCC Epidemiology). J Gastrointest Cancer. 2017 Sep 19;48(3):238–240. 28626852 10.1007/s12029-017-9959-0

[5] Garrido A, Djouder N. Cirrhosis: a questioned risk factor for hepatocellular carcinoma. Trends Cancer. 2021;7(1):29–36. doi: 10.1016/j.trecan.2020.08.005 32917550

[6] Ikeda K, Arase Y, Saitoh S, et al. Interferon beta prevents recurrence of hepatocellular carcinoma after complete resection or ablation of the primary tumor—A prospective randomized study of hepatitis C virus–related liver cancer. Hepatology. 2000;32(2):228–232. doi: 10.1053/jhep.2000.9409 10915728

[7] Acharya SK. Epidemiology of hepatocellular carcinoma in India. J Clin Exp Hepatol. 2014 Aug;4:S27–33. doi: 10.1016/j.jceh.2014.05.013 PMC4284206 25755607

[8] Liu CJ, Kao JH. Hepatitis B virus-related hepatocellular carcinoma: epidemiology and pathogenic role of viral factors. J Chin Med Assoc. 2007 Apr;70(4):141–145. doi: 10.1016/S1726-4901(09)70346-6 17475593

[9] Verme G, Brunetto MR, Oliveri F, et al. Role of hepatitis delta virus infection in hepatocellular carcinoma. Dig Dis Sci. 1991 Aug;36(8):1134–1136. doi: 10.1007/BF01297460 1650690

[10] Baffy G, Brunt EM, Caldwell SH. Hepatocellular carcinoma in non-alcoholic fatty liver disease: an emerging menace. J Hepatol. 2012 Jun;56(6):1384–1391. doi: 10.1016/j.jhep.2011.10.027 22326465

[11] Mancebo A, González–Diéguez ML, Cadahía V, et al. Annual incidence of hepatocellular carcinoma among patients with alcoholic cirrhosis and identification of risk groups. Clin Gastroenterol Hepatol. 2013 Jan;11(1):95–101. doi: 10.1016/j.cgh.2012.09.007 22982095

[12] Heckley GA, Jarl J, Asamoah BO, et al. How the risk of liver cancer changes after alcohol cessation: a review and meta-analysis of the current literature. BMC Cancer. 2011 Dec 13;11(1):446. doi: 10.1186/1471-2407-11-446 21995442 PMC3229519

[13] DWH H, RCL L, Chan LK, et al. Molecular pathogenesis of hepatocellular carcinoma. Liver Cancer. 2016;5(4):290–302. doi: 10.1159/000449340 27781201 PMC5075835

[14] Shibata T, Aburatani H. Exploration of liver cancer genomes. Nat Rev Gastroenterol Hepatol. 2014 Jun 28;11(6):340–349. 24473361 10.1038/nrgastro.2014.6

[15] Yoon SK. Molecular mechanism of hepatocellular carcinoma. Hepatoma Res. 2018;4(8):42. doi: 10.20517/2394-5079.2018.23

[16] Mazzanti R, Gramantieri L, Bolondi L. Hepatocellular carcinoma: epidemiology and clinical aspects. Mol Aspect Med. 2008;29(1–2):130–143. doi: 10.1016/j.mam.2007.09.008 18061252

[17] Campbell C, Wang T, McNaughton AL, et al. Risk factors for the development of hepatocellular carcinoma (HCC) in chronic hepatitis B virus (HBV) infection: a systematic review and meta-analysis. J Viral Hepat. 2021;28(3):493–507. doi: 10.1111/jvh.13452 33305479 PMC8581992

[18] Lampertico P, Agarwal K, Berg T, et al. EASL 2017 clinical practice guidelines on the management of hepatitis B virus infection. J Hepatol. 2017;67(2):370–398. doi: 10.1016/j.jhep.2017.03.021 28427875

[19] Wang N, Wang S, Li MY, et al. Cancer stem cells in hepatocellular carcinoma: an overview and promising therapeutic strategies. Therapeut Adv Med Oncol. 2018;10:175883591881628. doi: 10.1177/1758835918816287 PMC6304707 30622654

[20] Mishra L, Banker T, Murray J, et al. Liver stem cells and hepatocellular carcinoma. Hepatology. 2009;49(1):318–329. doi: 10.1002/hep.22704 19111019 PMC2726720

[21] Wu Y, Zhang J, Zhang X, et al. Cancer stem cells: a potential breakthrough in HCC-Targeted therapy. Front Pharmacol. 2020 [cited 2020 Mar 6];11:198. doi: 10.3389/fphar.2020.00198 32210805 PMC7068598

[22] Nio K, Yamashita T, Kaneko S. The evolving concept of liver cancer stem cells. Mol Cancer. 2017 Dec 30;16(1):4. 28137313 10.1186/s12943-016-0572-9 PMC5282887

[23] Fang X, Yan Q, Liu S, et al. Cancer stem cells in hepatocellular carcinoma: intrinsic and Extrinsic molecular mechanisms in Stemness regulation. Int J Mol Sci. 2022 Oct 14;23(20):12327. doi: 10.3390/ijms232012327 36293184 PMC9604119

[24] Benabou E, Salamé Z, Wendum D, et al. Insulin receptor isoform a favors tumor progression in human hepatocellular carcinoma by increasing stem/progenitor cell features. Cancer Lett. 2019 May;450:155–168. 30849481 10.1016/j.canlet.2019.02.037

[25] Mishra L, Banker T, Murray J, et al. Liver stem cells and hepatocellular carcinoma. Hepatology. 2009 Jan;49(1):318–329. doi: 10.1002/hep.22704 19111019 PMC2726720

[26] Nio K, Yamashita T, Kaneko S. The evolving concept of liver cancer stem cells. Mol Cancer. 2017;16(1). doi: 10.1186/s12943-016-0572-9 PMC5282887 28137313

[27] CKY N, Piscuoglio S, Terracciano LM. Molecular classification of hepatocellular carcinoma: the view from metabolic zonation. Hepatology. 2017 Nov 29;66(5):1377–1380. 28599064 10.1002/hep.29311

[28] Schulze K, Imbeaud S, Letouzé E, et al. Exome sequencing of hepatocellular carcinomas identifies new mutational signatures and potential therapeutic targets. Nat Genet. 2015 May 30;47(5):505–511. doi: 10.1038/ng.3252 25822088 PMC4587544

[29] Gómez-Sintes R, Arias E. Chaperone-mediated autophagy and disease: implications for cancer and neurodegeneration. Mol Aspects Med. 2021;82:82. doi: 10.1016/j.mam.2021.101025 PMC8711233 34629183

[30] Waly Raphael S, Yangde Z, YuXiang C. Hepatocellular carcinoma: focus on different aspects of management. ISRN Oncol. 2012;2012:1–12. doi: 10.5402/2012/421673 PMC3359687 22655206

[31] Moeini A, Cornellà H, Villanueva A. Emerging signaling pathways in hepatocellular carcinoma. Liver Cancer. 2012;1(2):83–93. doi: 10.1159/000342405 24159576 PMC3747540

[32] Sia D, Villanueva A. Signaling pathways in hepatocellular carcinoma. Oncology. 2011;81(Suppl. 1):18–23. doi: 10.1159/000333254 22212931

[33] Ngo MHT, Jeng HY, Kuo YC, et al. The role of IGF/IGF-1R signaling in hepatocellular carcinomas: stemness-related properties and drug resistance. Int J Mol Sci. 2021 Feb 16;22(4):1931. doi: 10.3390/ijms22041931 33669204 PMC7919800

[34] Wang H, Rao B, Lou J, et al. The function of the HGF/c-Met axis in hepatocellular carcinoma. Front Cell Dev Biol. 2020 Feb 7;8. doi:10.3389/fcell.2020.00055 PMC7018668 32117981

[35] Wang Z, Chen K, Jia Y, et al. Dual ARID1A/ARID1B loss leads to rapid carcinogenesis and disruptive redistribution of BAF complexes. Nat Cancer. 2020 Sep 7;1(9):909–922. doi: 10.1038/s43018-020-00109-0 34386776 PMC8357309

[36] Wang MD, Xiang H, Zhang L, et al. Integration of OV6 expression and CD68+ tumor-associated macrophages with clinical features better predicts the prognosis of patients with hepatocellular carcinoma. Transl Oncol. 2022 Nov;25:101509. 36030750 10.1016/j.tranon.2022.101509 PMC9428913

[37] Zhao C, Feng Q, Dou Z, et al. Local targeted therapy of liver metastasis from colon cancer by galactosylated liposome encapsulated with doxorubicin. PLoS One. 2013 Sep 11;8(9):e73860. doi: 10.1371/journal.pone.0073860 24040096 PMC3770687

[38] Kumar V, Rahman M, Gahtori P, et al. Current status and future directions of hepatocellular carcinoma-targeted nanoparticles and nanomedicine. Expert Opin Drug Delivery. 2021;18(6):673–694. doi: 10.1080/17425247.2021.1860939 33295218

[39] Guan MC, Ouyang W, Wang MD, et al. Biomarkers for hepatocellular carcinoma based on body fluids and feces. World J Gastrointest Oncol. 2021;13(5): doi: 10.4251/wjgo.v13.i5.351 PMC8131906 34040698

[40] Ahn JC, Teng PC, Chen PJ, et al. Detection of circulating tumor cells and their implications as a biomarker for diagnosis, prognostication, and therapeutic monitoring in hepatocellular carcinoma. Hepatology. 2021;73(1):422–436. doi: 10.1002/hep.31165 32017145 PMC8183673

[41] Zhang Q, Rong Y, Yi K, et al. Circulating tumor cells in hepatocellular carcinoma: single-cell based analysis, preclinical models, and clinical applications. Theranostics. 2020;10(26):12060–12071. doi: 10.7150/thno.48918 33204329 PMC7667686

[42] Jin X, Cai C, Qiu Y. Diagnostic value of circulating microRnas in hepatitis B virus-related hepatocellular carcinoma: a systematic review and meta-analysis. J Cancer. 2019;10(20):4754–4764. doi: 10.7150/jca.32833 31598147 PMC6775527

[43] Zhao XF, Li N, Lin DD, et al. Circulating MicroRNA-122 for the diagnosis of hepatocellular carcinoma: a meta-analysis. Biomed Res Int. 2020;2020: doi: 10.1155/2020/5353695 PMC7139899 32309434

[44] Qu J, Yang J, Chen M, et al. MicroRNA-21 as a diagnostic marker for hepatocellular carcinoma: a systematic review and meta-analysis. Pak J Med Sci. 2019;35(5): doi: 10.12669/pjms.35.5.685 PMC6717466 31489028

[45] Zeng Z, Dong J, Li Y, et al. The expression level and diagnostic value of microRNA-22 in HCC patients. Artif Cells Nanomed Biotechnol. 2020;48(1):683–686. doi: 10.1080/21691401.2019.1703723 32088997

[46] Pascut D, Cavalletto L, Pratama MY, et al. Serum miRNA are promising biomarkers for the detection of early hepatocellular carcinoma after treatment with direct-acting antivirals. Cancers (Basel). 2019;11(11):1773. doi: 10.3390/cancers11111773 31717959 PMC6895878

[47] Pratama MY, Visintin A, Crocè LS, et al. Circulatory miRNA as a biomarker for therapy response and disease-free survival in hepatocellular carcinoma. Cancers (Basel). 2020;12(10):2810. doi: 10.3390/cancers12102810 33003646 PMC7601056

[48] Liu C, Zhou X, Long Q, et al. Small extracellular vesicles containing miR-30a-3p attenuate the migration and invasion of hepatocellular carcinoma by targeting SNAP23 gene. Oncogene. 2021;40(2):233–245. doi: 10.1038/s41388-020-01521-7 33110233

[49] Oh BK, Kim H, Park HJ, et al. DNA methyltransferase expression and DNA methylation in human hepatocellular carcinoma and their clinicopathological correlation. Int J Mol Med. 2007;20(1): doi: 10.3892/ijmm.20.1.65 17549390

[50] Lin CH, Hsieh SY, Sheen IS, et al. Genome-wide hypomethylation in hepatocellular carcinogenesis. Cancer Res. 2001;61(10):4238–4243. 11358850

[51] Huang SL, Wang YM, Wang QY, et al. Mechanisms and clinical trials of hepatocellular carcinoma immunotherapy. Frontiers In Genetics. 2021;12. 2021 Front genet. 10.3389/fgene.2021.691391 PMC8296838 34306031

[52] Sangro B, Sarobe P, Hervás-Stubbs S, et al. Advances in immunotherapy for hepatocellular carcinoma. Nat Rev Gastroenterol Hepatol. 2021 Aug 13;18(8):525–543. doi: 10.1038/s41575-021-00438-0 33850328 PMC8042636

[53] Laskowski T, Rezvani K. Adoptive cell therapy: living drugs against cancer. J Exp Med. 2020 Dec 7;217(12). doi: 10.1084/jem.20200377 PMC7686916 33227136

[54] Gao X, Mi Y, Guo N, et al. Cytokine-induced killer cells as pharmacological tools for cancer immunotherapy. Front Immunol. 2017 Jul 6;8. doi: 10.3389/fimmu.2017.00774. PMC5498561 28729866

[55] Topalian SL, Muul LM, Solomon D, et al. Expansion of human tumor infiltrating lymphocytes for use in immunotherapy trials. J Immunol Methods. 1987 Aug;102(1):127–141. doi: 10.1016/S0022-1759(87)80018-2 3305708

[56] Ma W, Chen X, Yuan Y. T-cell-associated immunotherapy: a promising strategy for the treatment of hepatocellular carcinoma. Immunotherapy. 2017 Jun;9(7):523–525. doi: 10.2217/imt-2017-0053 28595519

[57] Jiang SS, Tang Y, Zhang YJ, et al. A phase I clinical trial utilizing autologous tumor-infiltrating lymphocytes in patients with primary hepatocellular carcinoma. Oncotarget. 2015 Dec 1;6(38):41339–41349. doi: 10.18632/oncotarget.5463 26515587 PMC4747409

[58] First CAR-T therapy to target BCMA gets FDA nod. Nat Biotechnol. 2021 May 12;39(5):531–531. doi:10.1038/s41587-021-00929-0 33981079

[59] Sun H, Sun C, Tian Z, et al. NK cells in immunotolerant organs. Cell Mol Immunol. 2013 May 8;10(3):202–212. doi: 10.1038/cmi.2013.9 23563087 PMC4012777

[60] Kalathil SG, Thanavala Y. Natural killer cells and T cells in hepatocellular carcinoma and viral hepatitis: Current status and perspectives for future immunotherapeutic approaches. Cells. 2021 May 28;10(6):1332. 34071188 10.3390/cells10061332 PMC8227136

[61] King C. CAR NK cell therapy for T follicular helper cells. Cell Rep Med. 2020 Apr;1(1):100009. doi: 10.1016/j.xcrm.2020.100009 33205057 PMC7659502

[62] Watanabe K, Nishikawa H. Engineering strategies for broad application of TCR-T- and CAR-T-cell therapies. Int Immunol. 2021 Oct 29;33(11):551–562. 34374779 10.1093/intimm/dxab052

[63] Werner M, Almer S, Prytz H, et al. Hepatic and extrahepatic malignancies in autoimmune hepatitis. A long-term follow-up in 473 Swedish patients. J Hepatol. 2009;50(2):388–393. doi: 10.1016/j.jhep.2008.08.022 19070390

[64] Béland K, Lapierre P, Alvarez F. Influence of genes, sex, age and environment on the onset of autoimmune hepatitis. World J Gastroenterol. 2009;15(9):1025. doi: 10.3748/wjg.15.1025 19266593 PMC2655185

[65] Granito A, Muratori L, Lalanne C, et al. Hepatocellular carcinoma in viral and autoimmune liver diseases: role of CD4+ CD25+ Foxp3+ regulatory T cells in the immune microenvironment. World J Gastroenterol. 2021;27(22):2994–3009. doi: 10.3748/wjg.v27.i22.2994 34168403 PMC8192285

[66] Philips CA, Rajesh S, Nair DC, et al. Hepatocellular carcinoma in 2021: an exhaustive update. Cureus. 2021. doi: 10.7759/cureus.19274 PMC8569837 34754704

[67] Webb GJ, Hirschfield GM, Krawitt EL, et al. Cellular and molecular mechanisms of autoimmune hepatitis. Annu Rev Pathol. 2018;13(1):247–292. doi: 10.1146/annurev-pathol-020117-043534 29140756

[68] Fedlu M. Aflatoxin and its public Health significance: a review. J Dairy Vet Sci. 2019;12(3). doi: 10.19080/JDVS.2019.12.555837

[69] Torres MCP, Bodini G, Furnari M, et al. Surveillance for hepatocellular carcinoma in patients with non-alcoholic fatty liver disease: universal or selective? Cancers. 2020;12(6):1422. doi: 10.3390/cancers12061422 32486355 PMC7352281

[70] Devarbhavi H, Asrani SK, Arab JP, et al. Global burden of liver disease: 2023 update. J Hepatol. 2023 Mar;79(2):516–537. doi: 10.1016/j.jhep.2023.03.017 36990226

[71] Fanning GC, Zoulim F, Hou J, et al. Therapeutic strategies for hepatitis B virus infection: towards a cure. Nat Rev Drug Discov. 2019;18(11):827–844. doi: 10.1038/s41573-019-0037-0 31455905

[72] Roca Suarez AA, Testoni B, Zoulim F. HBV 2021: new therapeutic strategies against an old foe. Liver Int. 2021;41(S1):15–23. doi: 10.1111/liv.14851 34155787

[73] Werle-Lapostolle B, Bowden S, Locarnini S, et al. Persistence of cccDNA during the natural history of chronic hepatitis B and decline during adefovir dipivoxil therapy1. Gastroenterology. 2004;126(7):1750–1758. doi: 10.1053/j.gastro.2004.03.018 15188170

[74] Ng SA, Lee C. Hepatitis B virus X gene and hepatocarcinogenesis. J Gastroenterol. 2011;46(8):974–990. doi: 10.1007/s00535-011-0415-9 21647825

[75] Shlomai A, de Jong YP, Rice CM. Virus associated malignancies: the role of viral hepatitis in hepatocellular carcinoma. Semin Cancer Biol. 2014 Jun;26:78–88. doi: 10.1016/j.semcancer.2014.01.004 24457013 PMC4048791

[76] Jiang Y, Han Q, Zhao H, et al. The mechanisms of HBV-Induced hepatocellular carcinoma. J Hepatocell Carcinoma. 2021;8:435–450. doi: 10.2147/JHC.S307962 34046368 PMC8147889

[77] Ng KY, Chai S, Tong M, et al. C-terminal truncated hepatitis B virus X protein promotes hepatocellular carcinogenesis through induction of cancer and stem cell-like properties. Oncotarget. 2016 Apr 26;7(17):24005–24017. doi: 10.18632/oncotarget.8209 27006468 PMC5029680

[78] Murakami S. Hepatitis B virus X protein: a multifunctional viral regulator. J Gastroenterol. 2001 Oct 1;36(10):651–660. 11686474 10.1007/s005350170027

[79] Martin-Vilchez S, Lara-Pezzi E, Trapero-Marugán M, et al. The molecular and pathophysiological implications of hepatitis B X antigen in chronic hepatitis B virus infection. Rev Med Virol. 2011 Sep;21(5):315–329. doi: 10.1002/rmv.699 21755567

[80] Ding WB, Wang MC, Yu J, et al. Hbv/pregenomic RNA increases the stemness and promotes the development of HBV-Related HCC through reciprocal regulation with Insulin-like growth factor 2 mRNA-binding protein 3. Hepatology. 2021;74(3):1480–1495. doi: 10.1002/hep.31850 33825218

[81] Lamouille S, Xu J, Derynck R. Molecular mechanisms of epithelial–mesenchymal transition. Nat Rev Mol Cell Biol. 2014;15(3):178–196. doi: 10.1038/nrm3758 24556840 PMC4240281

[82] Kim GW, Imam H, Khan M, et al. HBV-Induced increased N6 methyladenosine modification of PTEN RNA affects innate immunity and contributes to HCC. Hepatology. 2021;73(2):533–547. doi: 10.1002/hep.31313 32394474 PMC7655655

[83] Seo SI, Kim HS, Kim WJ, et al. Diagnostic value of PIVKA-II and alpha-fetoprotein in hepatitis b virus-associated hepatocellular carcinoma. World J Gastroenterol. 2015;21(13):3928. doi: 10.3748/wjg.v21.i13.3928 25852278 PMC4385540

[84] Li X, Guo Y, Wang X, et al. Clinical significance of serum miR-487b in HBV-related hepatocellular carcinoma and its potential mechanism. Infect Dis. 2021;53(7):546–554. doi: 10.1080/23744235.2021.1901981 33783293

[85] de Mattos ÂZ, Debes JD, Boonstra A, et al. Current impact of viral hepatitis on liver cancer development: the challenge remains. World J Gastroenterol. 2021;27(24):3556–3567. doi: 10.3748/wjg.v27.i24.3556 34239269 PMC8240060

[86] Wungu CDK, Ariyanto FC, Prabowo GI, et al. Meta-analysis: association between hepatitis B virus preS mutation and hepatocellular carcinoma risk. J Viral Hepat. 2021;28(1):61–71. doi: 10.1111/jvh.13402 32896077

[87] Rizzo GEM, Cabibbo G, Craxì A. Hepatitis B virus-associated hepatocellular carcinoma. Viruses. 2022 May 7;14(5):986. 35632728 10.3390/v14050986 PMC9146458

[88] Wang LHC, Huang W, Lai MD, et al. Aberrant cyclin a expression and centrosome overduplication induced by hepatitis B virus pre-S2 mutants and its implication in hepatocarcinogenesis. Carcinogenesis. 2012;33(2):466–472. doi: 10.1093/carcin/bgr296 22159224

[89] Shuyao W, Mingyang B, Feifei M, et al. CK19 predicts recurrence and prognosis of HBV positive HCC. J Gastrointestinal Surg. 2021;26(2):341–351. doi: 10.1007/s11605-021-05107-w 34506016

[90] De Battista D, Zamboni F, Gerstein H, et al. Molecular signature and immune landscape of HCV-Associated hepatocellular carcinoma (HCC): differences and similarities with HBV-HCC. J Hepatocell Carcinoma. 2021;8:1399–1413. doi: 10.2147/JHC.S325959 34849372 PMC8615147

[91] Trehanpati N, Vyas AK. Immune regulation by T regulatory cells in hepatitis B virus-related inflammation and cancer. Scand J Immunol. 2017 Mar;85(3):175–181. doi: 10.1111/sji.12524 28109025

[92] Lim CJ, Lee YH, Pan L, et al. Multidimensional analyses reveal distinct immune microenvironment in hepatitis B virus-related hepatocellular carcinoma. Gut. 2019 May;68(5):916–927. doi: 10.1136/gutjnl-2018-316510 29970455

[93] Wei T, Zhang LN, Lv Y, et al. Overexpression of platelet-derived growth factor receptor alpha promotes tumor progression and indicates poor prognosis in hepatocellular carcinoma. Oncotarget. 2014 Nov 15;5(21):10307–10317. doi: 10.18632/oncotarget.2537 25333264 PMC4279374

[94] Fu J, Xu D, Liu Z, et al. Increased regulatory T cells correlate with CD8 T-Cell impairment and poor survival in hepatocellular carcinoma patients. Gastroenterology. 2007 Jun;132(7):2328–2339. doi: 10.1053/j.gastro.2007.03.102 17570208

[95] Yang Y, Han Q, Hou Z, et al. Exosomes mediate hepatitis B virus (HBV) transmission and NK-cell dysfunction. Cell Mol Immunol. 2017 May 30;14(5):465–475. doi: 10.1038/cmi.2016.24 27238466 PMC5423088

[96] Wu Y, Hao X, Wei H, et al. Blockade of T‐cell receptor with ig and ITIM domains elicits potent antitumor immunity in naturally occurring HBV‐related HCC in mice. Hepatology. 2023 Mar;77(3):965–981. doi: 10.1002/hep.32715 35938354

[97] Martin P, Nguyen MH, Dieterich DT, et al. Treatment algorithm for managing chronic hepatitis B virus infection in the United States: 2021 update. Clin Gastroenterol Hepatol. 2021;20(8):1766–1775. doi: 10.1016/j.cgh.2021.07.036 34329775

[98] Riazi K, Azhari H, Charette JH, et al. The prevalence and incidence of NAFLD worldwide: a systematic review and meta-analysis. Lancet Gastroenterol Hepatol. 2022 Sep;7(9):851–861. doi: 10.1016/S2468-1253(22)00165-0 35798021

[99] Kucukoglu O, Sowa JP, Mazzolini GD, et al. Hepatokines and adipokines in NASH-related hepatocellular carcinoma. J Hepatol. 2021;74(2):442–457. doi: 10.1016/j.jhep.2020.10.030 33161047

[100] Mittal S, El-Serag HB, Sada YH, et al. Hepatocellular carcinoma in the absence of cirrhosis in United States veterans is associated with nonalcoholic fatty liver disease. Clin Gastroenterol Hepatol. 2016;14(1):124–131.e1. doi: 10.1016/j.cgh.2015.07.019 26196445 PMC4690789

[101] Tsuneto A, Hida A, Sera N, et al. Fatty liver incidence and predictive variables. Hypertens Res. 2010;33(6):638–643. doi: 10.1038/hr.2010.45 20379184

[102] Ghouri YA, Mian I, Rowe JH. Review of hepatocellular carcinoma: epidemiology, etiology, and carcinogenesis. J Carcinog. 2017;16(1):1. doi: 10.4103/jcar.JCar_9_16 28694740 PMC5490340

[103] Myers S, Neyroud-Caspar I, Spahr L, et al. NAFLD and MAFLD as emerging causes of HCC: a populational study. JHEP Rep. 2021;3(2):100231. doi: 10.1016/j.jhepr.2021.100231 33748726 PMC7957147

[104] Zhang DY, Friedman SL. Fibrosis-dependent mechanisms of hepatocarcinogenesis. Hepatology. 2012;56(2):769–775. doi: 10.1002/hep.25670 22378017 PMC4087159

[105] Mantovani A, Targher G. Type 2 diabetes mellitus and risk of hepatocellular carcinoma: spotlight on nonalcoholic fatty liver disease. Ann translat Med. 2017;5(13):270–270. doi: 10.21037/atm.2017.04.41 PMC5515814 28758096

[106] Michelotti A, de Scordilli M, Palmero L, et al. NAFLD-related hepatocarcinoma: the malignant side of metabolic syndrome. Cells. 2021;10(8):2034. doi: 10.3390/cells10082034 34440803 PMC8391372

[107] Eslam M, Sanyal AJ, George J, et al. MAFLD: a consensus-driven proposed nomenclature for metabolic associated fatty liver disease. Gastroenterology. 2020;158(7):1999–2014.e1. doi: 10.1053/j.gastro.2019.11.312 32044314

[108] Al-Serri A, Anstee QM, Valenti L, et al. The SOD2 C47T polymorphism influences NAFLD fibrosis severity: evidence from case-control and intra-familial allele association studies. J Hepatol. 2012;56(2):448–454. doi: 10.1016/j.jhep.2011.05.029 21756849

[109] Eldafashi N, Darlay R, Shukla R, et al. A pdcd1 role in the genetic predisposition to nafld-hcc? Cancers (Basel). 2021;13(6):1412. doi: 10.3390/cancers13061412 33808740 PMC8003582

[110] Margini C, Dufour JF. The story of HCC in NAFLD: from epidemiology, across pathogenesis, to prevention and treatment. Liver Int. 2016;36(3):317–324. doi: 10.1111/liv.13031 26601627

[111] Pirazzi C, Valenti L, Motta BM, et al. PNPLA3 has retinyl-palmitate lipase activity in human hepatic stellate cells. Hum Mol Genet. 2014;23(15):4077–4085. doi: 10.1093/hmg/ddu121 24670599 PMC4082369

[112] Eslam M, Valenti L, Romeo S. Genetics and epigenetics of NAFLD and NASH: clinical impact. J Hepatol. 2018;68(2):268–279. doi: 10.1016/j.jhep.2017.09.003 29122391

[113] Kriss M, Golden-Mason L, Kaplan J, et al. Increased hepatic and circulating chemokine and osteopontin expression occurs early in human NAFLD development. PLoS One. 2020;15(7):e0236353. doi: 10.1371/journal.pone.0236353 32730345 PMC7392333

[114] Michelotti GA, Machado MV, Diehl AM. NAFLD, NASH and liver cancer. Nat Rev Gastroenterol Hepatol. 2013;10(11):656–665. doi: 10.1038/nrgastro.2013.183 24080776

[115] Naik A, Košir R, Rozman D. Genomic aspects of NAFLD pathogenesis. Genomics. 2013;102(2):84–95. doi: 10.1016/j.ygeno.2013.03.007 23545492

[116] WKK W, Zhang L, Chan MTV. Autophagy, NAFLD and NAFLD-related HCC. Adv Exp Med Biol. 2018;1061:127–138. doi: 10.1007/978-981-10-8684-7_10 29956211

[117] Nair S, Mason A, Eason J, et al. Is obesity an independent risk factor for hepatocellular carcinoma in cirrhosis? Hepatology. 2002;36(1):150–155. doi: 10.1053/jhep.2002.33713 12085359

[118] Stefan N, Häring HU. The role of hepatokines in metabolism. Nat Rev Endocrinol. 2013;9(3):144–152. doi: 10.1038/nrendo.2012.258 23337953

[119] Adolph TE, Grander C, Grabherr F, et al. Adipokines and non-alcoholic fatty liver disease: multiple interactions. Int J Mol Sci. 2017;18(8):1649. doi: 10.3390/ijms18081649 28758929 PMC5578039

[120] Hoki T, Miyanishi K, Tanaka S, et al. Increased duodenal iron absorption through up-regulation of divalent metal transporter 1 from enhancement of iron regulatory protein 1 activity in patients with nonalcoholic steatohepatitis. Hepatology. 2015;62(3):751–761. doi: 10.1002/hep.27774 25753988

[121] Blaya D, Coll M, Rodrigo-Torres D, et al. Integrative microRNA profiling in alcoholic hepatitis reveals a role for microRNA-182 in liver injury and inflammation. Gut. 2016;65(9):1535–1545. doi: 10.1136/gutjnl-2015-311314 27196584

[122] Coulouarn C, Factor VM, Andersen JB, et al. Loss of miR-122 expression in liver cancer correlates with suppression of the hepatic phenotype and gain of metastatic properties. Oncogene. 2009;28(40):3526–3536. doi: 10.1038/onc.2009.211 19617899 PMC3492882

[123] Liu Z, Wang Y, Borlak J, et al. Mechanistically linked serum miRnas distinguish between drug induced and fatty liver disease of different grades. Sci Rep. 2016;6(1):6. doi: 10.1038/srep23709 27045805 PMC4820692

[124] Hsu SH, Wang B, Kota J, et al. Essential metabolic, anti-inflammatory, and anti-tumorigenic functions of miR-122 in liver. J Clin Investig. 2012;122(8):2871–2883. doi: 10.1172/JCI63539 22820288 PMC3408748

[125] He Y, Hwang S, Cai Y, et al. MicroRNA-223 ameliorates nonalcoholic steatohepatitis and cancer by targeting Multiple inflammatory and oncogenic genes in hepatocytes. Hepatology. 2019;70(4):1150–1167. doi: 10.1002/hep.30645 30964207 PMC6783322

[126] WHO. World Health organization | hepatitis C. Bulletin of the World Health organization - fact sheet N°164. 2020.

[127] Cabibbo G, Petta S, Barbàra M, et al. A meta-analysis of single HCV-untreated arm of studies evaluating outcomes after curative treatments of HCV-related hepatocellular carcinoma. Liver Int. 2017;37(8):1157–1166. doi: 10.1111/liv.13357 28061016

[128] Singal AG, Rich NE, Mehta N, et al. Direct-acting antiviral therapy for hepatitis C virus infection is associated with increased survival in patients with a history of hepatocellular carcinoma. Gastroenterology. 2019;157(5):1253–1263.e2. doi: 10.1053/j.gastro.2019.07.040 31374215 PMC6815711

[129] Saito T, Sugimoto M, Okumoto K, et al. Serum metabolome profiles characterized by patients with hepatocellular carcinoma associated with hepatitis B and C. World J Gastroenterol. 2016;22(27):6224. doi: 10.3748/wjg.v22.i27.6224 27468212 PMC4945981

[130] Zhang Y. Detection of epigenetic aberrations in the development of hepatocellular carcinoma. Methods Mol Biol. 2015;1238:709–731. doi: 10.1007/978-1-4939-1804-1_37 25421688

[131] Ahumada A, Rayón L, Usón C, et al. Hepatocellular carcinoma risk after viral response in hepatitis C virus-advanced fibrosis: who to screen and for how long? World J Gastroenterol. 2021;27(40):6737–6749. doi: 10.3748/wjg.v27.i40.6737 34790004 PMC8567476

[132] Poynard T, Moussalli J, Munteanu M, et al. Slow regression of liver fibrosis presumed by repeated biomarkers after virological cure in patients with chronic hepatitis C. J Hepatol. 2013;59(4):675–683. doi: 10.1016/j.jhep.2013.05.015 23712051

[133] Hamdane N, Jühling F, Crouchet E, et al. HCV-Induced epigenetic changes associated with liver cancer risk persist after sustained virologic response. Gastroenterology. 2019;156(8):2313–2329.e7. doi: 10.1053/j.gastro.2019.02.038 30836093 PMC8756817

[134] Akkari L, Grégoire D, Floc’h N, et al. Hepatitis C viral protein NS5A induces EMT and participates in oncogenic transformation of primary hepatocyte precursors. J Hepatol. 2012;57(5):1021–1028. doi: 10.1016/j.jhep.2012.06.027 22750466

[135] Nault JC, Zucman-Rossi J. TERT promoter mutations in primary liver tumors. Clin Res Hepatol Gastroenterol. 2016;40(1):9–14. doi: 10.1016/j.clinre.2015.07.006 26336998

[136] Domovitz T, Gal-Tanamy M. Tracking down the epigenetic footprint of hcv-induced hepatocarcinogenesis. J Clin Med. 2021;10(3):551. doi: 10.3390/jcm10030551 33540858 PMC7867330

[137] Zhao P, Malik S, Xing S. Epigenetic mechanisms involved in HCV-Induced hepatocellular carcinoma (HCC). Front Oncol. 2021;11: doi: 10.3389/fonc.2021.677926 PMC8320331 34336665

[138] Zhao W, Liu H, Liu W, et al. Abnormal activation of the synuclein-gamma gene in hepatocellular carcinomas by epigenetic alteration. Int J Oncol. 2006;28(5): doi: 10.3892/ijo.28.5.1081 16596223

[139] Ke PY, Chen SSL. Activation of the unfolded protein response and autophagy after hepatitis C virus infection suppresses innate antiviral immunity in vitro. J Clin Investig. 2011;121(1):37–56. doi: 10.1172/JCI41474 21135505 PMC3007134

[140] Jassey A, Liu CH, Changou C, et al. Hepatitis C virus non-structural protein 5A (NS5A) disrupts mitochondrial dynamics and induces mitophagy. Cells. 2019;8(4):290. doi: 10.3390/cells8040290 30934919 PMC6523690

[141] Eslam M, Sarin SK, Wong VWS, et al. The Asian Pacific association for the study of the liver clinical practice guidelines for the diagnosis and management of metabolic associated fatty liver disease. Hepatol Int. 2020;14(6):889–919. Hepatology International. doi: 10.1007/s12072-020-10094-2 33006093

[142] Chan AWH, Wong GLH, Chan HY, et al. Concurrent fatty liver increases risk of hepatocellular carcinoma among patients with chronic hepatitis B. J Gastroenterol Hepatol (Australia). 2017;32(3):667–676. doi: 10.1111/jgh.13536 27547913

[143] Vongsuvanh R, van der Poorten D, George J. Non-alcoholic fatty liver disease-related hepatocellular carcinoma: a sleeping tiger in the asia pacific. Hepatol Int. 2013;7(S2):823–832. doi: 10.1007/s12072-013-9453-0 26202297

[144] Febbraio MA, Reibe S, Shalapour S, et al. Preclinical models for studying NASH-Driven HCC: how useful are they? Cell Metab. 2019;29(1):18–26. doi: 10.1016/j.cmet.2018.10.012 30449681 PMC6326872

[145] Meroni M, Longo M, Fracanzani AL, et al. MBOAT7 down-regulation by genetic and environmental factors predisposes to MAFLD. EBioMedicine. 2020;57:102866. EBioMedicine. 2020. doi: 10.1016/j.ebiom.2020.102866. 32629394 PMC7339032

[146] Chen S, Guo H, Xie M, et al. Neutrophil: an emerging player in the occurrence and progression of metabolic associated fatty liver disease. Int Immunopharmacol. 2021;97:107609. doi: 10.1016/j.intimp.2021.107609 33887577

[147] Zhou SL, Zhou ZJ, Hu ZQ, et al. Tumor-associated neutrophils recruit macrophages and T-Regulatory cells to promote progression of hepatocellular carcinoma and resistance to Sorafenib. Gastroenterology. 2016;150(7):1646–1658.e17. doi: 10.1053/j.gastro.2016.02.040 26924089

[148] Pelusi S, Baselli G, Pietrelli A, et al. Rare pathogenic variants predispose to hepatocellular carcinoma in nonalcoholic fatty liver disease. Sci Rep. 2019;9(1): doi: 10.1038/s41598-019-39998-2 PMC6403344 30842500

[149] Ally A, Balasundaram M, Carlsen R, et al. Comprehensive and integrative genomic characterization of hepatocellular carcinoma. Cell. 2017;169(7):1327–1341.e23. doi: 10.1016/j.cell.2017.05.046 28622513 PMC5680778

[150] Abdel-Moety A, Baddour N, Salem P, et al. SQSTM1 expression in hepatocellular carcinoma and relation to tumor recurrence after radiofrequency ablation. J Clin Exp Hepatol. 2022 May;12(3):774–784. doi: 10.1016/j.jceh.2021.12.001 35677515 PMC9168718

[151] Stumptner C, Fuchsbichler A, Zatloukal K, et al. In vitro production of Mallory bodies and intracellular hyaline bodies: the central role of sequestosome 1/p62. Hepatology. 2007;46(3):851–860. doi: 10.1002/hep.21744 17685470

[152] Sze KM, Ho DW, Chiu Y, et al. Hepatitis B virus–telomerase reverse transcriptase promoter integration harnesses host ELF4, resulting in telomerase reverse transcriptase gene transcription in hepatocellular carcinoma. Hepatology. 2021 Jan 26;73(1):23–40. doi: 10.1002/hep.31231 32170761 PMC7898544

[153] Cortes E, Lachowski D, Rice A, et al. Retinoic acid receptor‐β is downregulated in hepatocellular carcinoma and cirrhosis and its expression inhibits myosin‐driven activation and durotaxis in hepatic stellate cells. Hepatology. 2019 Feb 17;69(2):785–802. doi: 10.1002/hep.30193 30055117

[154] Kim H, Park J, Kim Y, et al. Serum fibronectin distinguishes the early stages of hepatocellular carcinoma. Sci Rep. 2017 Aug 25;7(1):9449. doi: 10.1038/s41598-017-09691-3 28842594 PMC5573357

[155] Jin ZL, PHXYYJDT. SUMO-specific protease SENP5 controls DNA damage response and promotes tumorigenesis in hepatocellular carcinoma. Eur Rev Med Pharmacol Sci. 2016 Sep;20(17):3566–3573. PMID: 27649656. 27649656

[156] Torimura T, Ueno T, Kin M, et al. Overexpression of angiopoietin-1 and angiopoietin-2 in hepatocellular carcinoma. J Hepatol. 2004 May;40(5):799–807. doi: 10.1016/j.jhep.2004.01.027 15094228

[157] Junjie X, Songyao J, Minmin S, et al. The association between Toll-like receptor 2 single-nucleotide polymorphisms and hepatocellular carcinoma susceptibility. BMC Cancer. 2012;12(1):12. doi: 10.1186/1471-2407-12-57 22309608 PMC3311588

[158] Zucman-Rossi J, Villanueva A, Nault JC, et al. Genetic landscape and biomarkers of hepatocellular carcinoma. Gastroenterology. 2015;149(5):1226–1239.e4. doi: 10.1053/j.gastro.2015.05.061 26099527

[159] Müller M, Bird TG, Nault JC. The landscape of gene mutations in cirrhosis and hepatocellular carcinoma. J Hepatol. 2020;72(5):990–1002. doi: 10.1016/j.jhep.2020.01.019 32044402

[160] Pocha C, Xie C. Hepatocellular carcinoma in alcoholic and non-alcoholic fatty liver disease—one of a kind or two different enemies? Transl Gastroenterol Hepatol. 2019;4:72–72. doi: 10.21037/tgh.2019.09.01 31728429 PMC6851438

[161] Nahon P, Sutton A, Rufat P, et al. Myeloperoxidase and superoxide dismutase 2 polymorphisms comodulate the risk of hepatocellular carcinoma and death in alcoholic cirrhosis. Hepatology. 2009;50(5):1484–1493. doi: 10.1002/hep.23187 19731237

[162] Jin F, Qu LS, Shen XZ. Association between C282Y and H63D mutations of the HFE gene with hepatocellular carcinoma in European populations: a meta-analysis. J Exp Clin Cancer Res. 2010;29(1). doi: 10.1186/1756-9966-29-18 PMC2845109 20196837

[163] Rambaldi A, Gluud C. S-adenosyl-L-methionine for alcoholic liver diseases. Cochrane Database Syst Rev. 2015;2015: doi: 10.1002/14651858.CD002235.pub3 16625556

[164] Donohue TM. Alcohol-induced steatosis in liver cells. World J Gastroenterol. 2007;13(37):4974. doi: 10.3748/wjg.v13.i37.4974 17854140 PMC4434621

[165] Lackner C, Tiniakos D. Fibrosis and alcohol-related liver disease. J Hepatol. 2019;70(2):294–304. doi: 10.1016/j.jhep.2018.12.003 30658730

[166] Roy N, Dasgupta D, Mukhopadhyay I, et al. Genetic association and gene-gene interaction reveal genetic variations in ADH1B, GSTM1 and MnSOD independently confer risk to alcoholic liver diseases in India. PLoS One. 2016;11(3):e0149843. doi: 10.1371/journal.pone.0149843 26937962 PMC4777485

[167] Nakano S, Eso Y, Okada H, et al. Recent advances in immunotherapy for hepatocellular carcinoma. Cancers. 2020;12(4):775. doi: 10.3390/cancers12040775 32218257 PMC7226090

